# Spatial proteomics reveals recombinant human laminin-111 restores adhesion signaling to laminin-**α**2–deficient muscle

**DOI:** 10.1172/jci.insight.194581

**Published:** 2025-10-16

**Authors:** Hailey J. Hermann, Ryan D. Wuebbles, Marisela Dagda, Axel Muñoz, Lauren L. Parker, Paula C. Guzman, Lola T. Byrne, Steven A. Moore, Dean J. Burkin

**Affiliations:** 1 Department of Pharmacology, Reno School of Medicine, University of Nevada, Reno, Nevada, USA.; 2 Department of Pathology, Carver College of Medicine, University of Iowa, Iowa City, Iowa, USA.

**Keywords:** Cell biology, Muscle biology, Extracellular matrix, Genetic diseases, Proteomics

## Abstract

Laminin-α2–related congenital muscular dystrophy (LAMA2-CMD) is a severe neuromuscular disorder caused by mutations in the *LAMA2* gene, leading to loss of heterotrimers laminin-211/221, key components of the skeletal muscle extracellular matrix. Their absence disrupts adhesion between the cytoskeleton and extracellular matrix, resulting in progressive muscle wasting. Laminin-211/221 interacts with adhesion complexes such as the dystrophin/utrophin glycoprotein complex and α7β1-integrin. However, the regulatory mechanisms of these laminin-binding complexes and the broader role of laminin’s influence on the formation of the macromolecular network in skeletal muscle remain unclear. We previously demonstrated that delivering mouse laminin-111 to the *dy^W–/–^* mouse model of LAMA2-CMD prevented disease progression, improved strength, and extended survival. We hypothesize that laminin-111, the embryonic laminin isoform, restores key adhesion-signaling networks. Using spatial proteomics on patient and mouse muscle, we identified loss of essential signaling components: heat shock proteins 27 and 70, c-Jun N-terminal kinase, and glucose transporter 1 in laminin-α2–deficient muscle. Treatment with recombinant human laminin-111 (rhLAM-111) restored protein localization, reduced ROS, and promoted glycolytic, prosurvival signaling. These findings highlight laminin’s role in maintaining muscle homeostasis and metabolism and support the therapeutic potential of rhLAM-111 for treating LAMA2-CMD by restoring adhesion and intracellular signaling in dystrophic muscle.

## Abstract 2

Laminin-α2-related congenital muscular dystrophy is a fatal neuromuscular disorder. Spatial-omics revealed that recombinant human laminin-111 treatment rescued signaling complexes in laminin-α2 deficient muscle.

## Graphical abstract

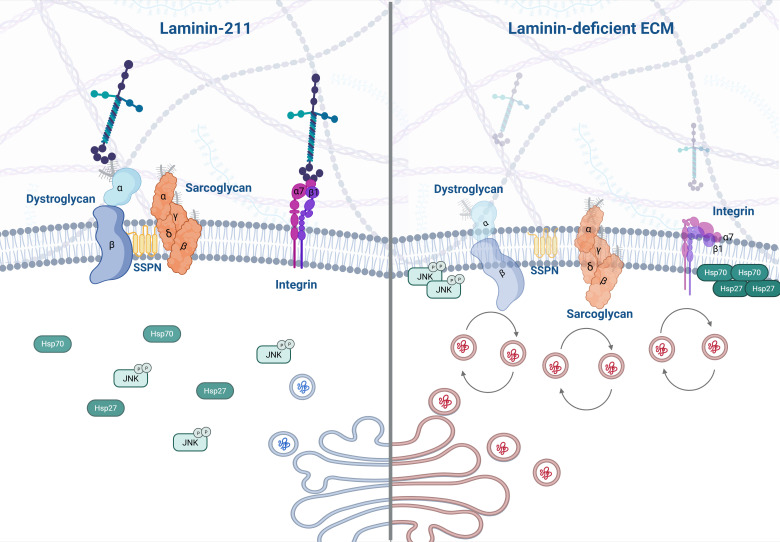

## Introduction

Congenital muscular dystrophies (CMDs) represent a diverse range of early-onset neuromuscular disorders marked by delayed motor development, hypotonia, and extensive muscle degeneration (1). Laminin-α2–related CMD (LAMA2-CMD) accounts for a quarter of CMD cases, with an estimated prevalence of 1.36–20 cases per million (2). LAMA2-CMD is caused by mutations in the *LAMA2* gene that lead to a truncated or complete loss of the laminin-α2 chain (3, 4). The absence of laminin-211 and 221 heterotrimers results in the loss of cytoskeleton–basal lamina interactions, leading to sarcolemma instability and activation of apoptotic pathways (4). Failed muscle regeneration is a hallmark in laminin-α2–deficient muscle, indicating the importance of a laminin-211/221–rich microenvironment for maintaining satellite cells and promoting myogenic repair (5, 6). As the disease progresses, patients with LAMA2-CMD lose ambulation and develop respiratory insufficiency, ultimately leading to premature death (7). Currently, there are no effective therapeutics, and the only form of treatment is symptomatic care management (8).

Laminin-111, the predominant laminin isoform in embryonic skeletal muscle, is structurally and functionally similar to laminin-211/221 (9). We have previously shown that administering mouse laminin-111 to the muscles of the *dy^W–/–^* mouse model of LAMA2-CMD can impede disease progression and enhance muscle strength (10–12). Several studies have shown that laminin-111 is an effective protein therapy in preclinical models of Duchenne muscular dystrophy (DMD) and *dy^W–/–^* mice (13–15). The expression of laminin-111 in many adult tissues, including the kidney and brain, will likely minimize the risk of an immune response to recombinant protein therapeutic intervention using recombinant human laminin-111 (rhLAM-111), unlike direct replacement with laminin-211 (12, 16). Therefore, in patients with LAMA2-CMD, rhLAM-111 is likely to be a suitable protein replacement alternative to rhLAM-211.

Severe myonecrosis with accompanying myophagocytosis, regeneration, and endomysial fibrosis are observed in LAMA2-CMD, particularly in the initial stages of the disease (7). In *dy^W–/–^* mice, a similar degree of dystrophic muscle pathology is evident as early as 3 weeks of age (17). The complex pathological processes in LAMA2-CMD pose challenges for interpreting studies derived from Western blot or mass spectrometry. To address this issue, we used GeoMX digital spatial profiling (DSP) to isolate regions of interest (ROIs) within intact individual muscle fibers and whole areas of tissue, including myofibers and their surrounding microenvironment. This approach integrates histological and genomic methods, offering a multiplexed analysis of proteins within ROIs using photo-cleavable oligonucleotide tags attached to designated antibodies (18). By examining myofibers in LAMA2-CMD and mouse tissue, we compared signaling pathways, their activity and localization, and the effects of rhLAM-111 treatment on *dy^W–/–^* mouse muscle. This study utilizes quantitative spatial proteomics technology to investigate the molecular pathophysiology and potential corrective treatment in LAMA2-CMD. Our analyses revealed complex changes in cell signaling driven by laminin-adhesion complexes that impact muscle cell adhesion, proteostasis, inflammation, and metabolic function that are restored by treatment with rhLAM-111.

## Results

### DSP analysis of patients with LAMA2-CMD and controls.

**Figure 1:**
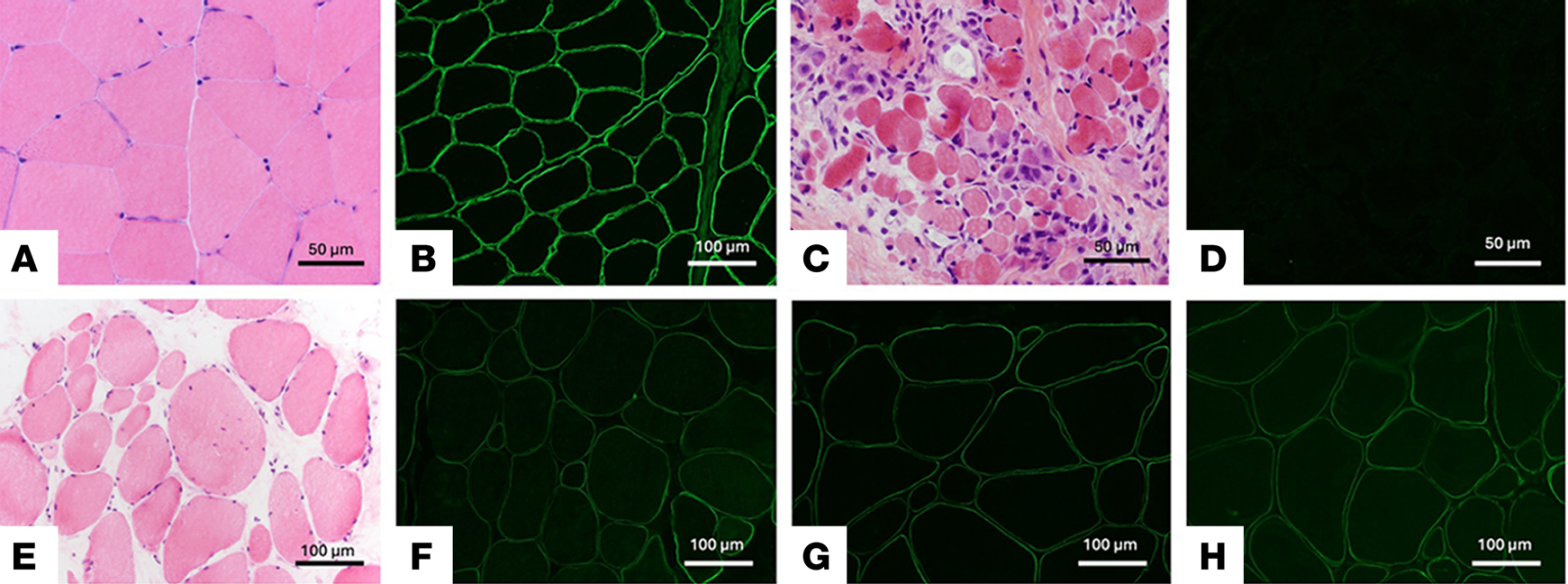
Histopathology and laminin-211 IF of human muscle biopsies. (**A**) H&E-stained cryosections are shown for normal skeletal muscle, and (**B**) laminin-211 IF stained cryosections of normal skeletal muscle. (**C**) H&E-stained cryosections for severe LAMA2-CMD (case 1) and (**D**) laminin-stained IF. (**E**) Mild, partially LAMA2-CMD (case 4) histopathology and (**F**–**H**) laminin-211 IF staining using antibodies against C-terminus, middle portion, and N-terminus of laminin-211. Scale bars: 50 μm (**A**, **C**, and **D**) and 100 μm (**B** and **E**–**H**).

**Figure 2:**
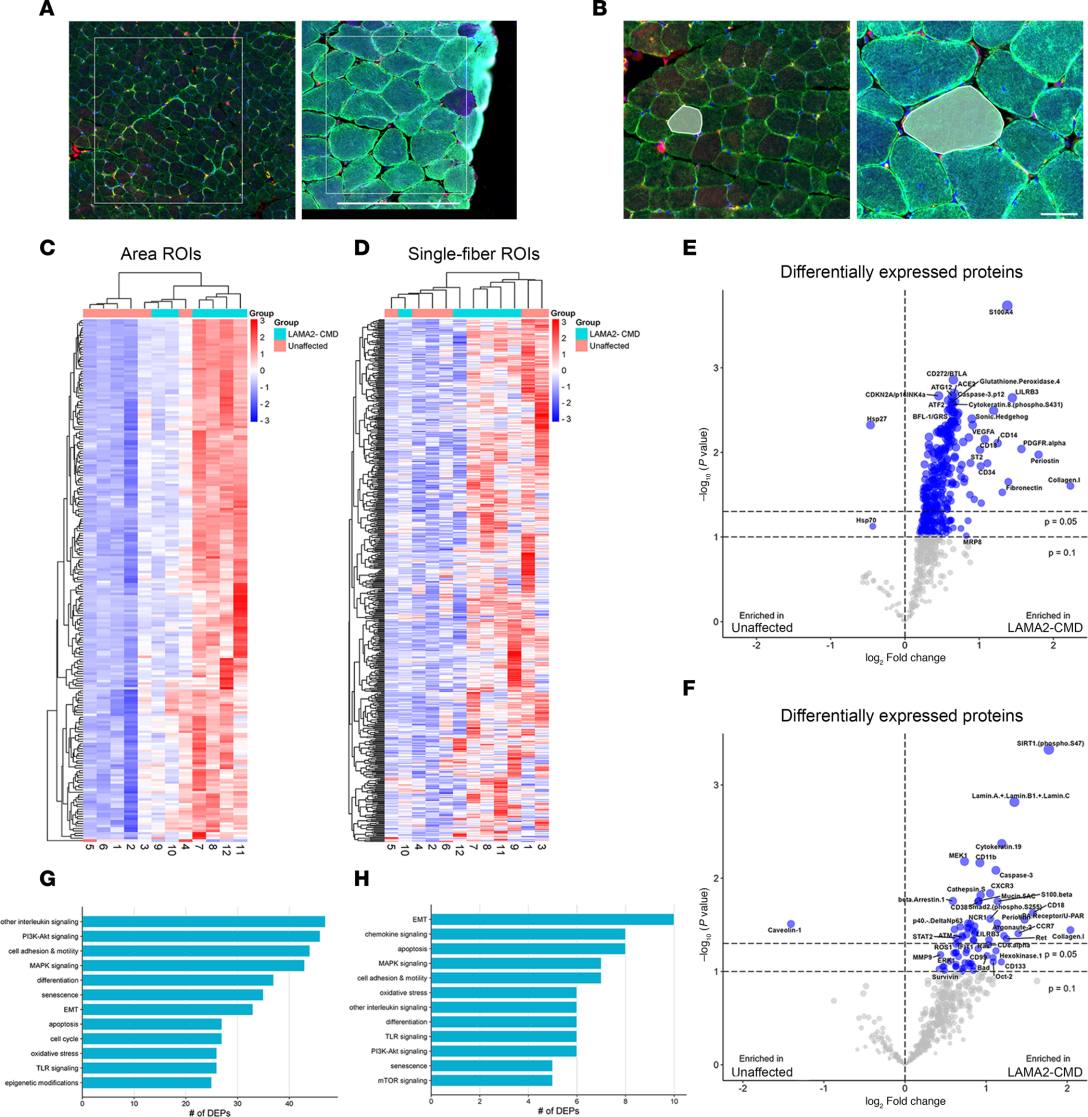
Spatial profiling reveals large-scale changes in LAMA2-CMD patient muscle. (**A**) Selected area ROIs from unaffected and patient tissues based on morphological markers DNA, desmin, CD31, and CD68. Scale bar: 500 μm. (**B**) Single-fiber ROIs from unaffected and patient tissues were selected based on desmin expression. Scale bar: 50 μm. (**C** and **D**) Protein expression heatmaps of significantly differentially expressed proteins (DEPs) from the GeoMx IPA dataset for the area (**C**) and single-fiber ROIs (**D**) (*P* < 0.05; 95% confidence interval). (**E** and **F**) Volcano plots showing DEPs in unaffected (left) and LAMA2-CMD (right) area ROIs (**E**) and single-fiber ROIs (**F**), determined by 2-tailed mixed effects linear regression with FDR multiple test correction. The top horizontal line indicates the cutoff for proteins with an adjusted *P* value of less than 0.05. (**G** and **H**) LAMA2-CMD patient DEPs sorted into biological processes based on NanoString’s target group classification for area (**G**) and single-fiber ROIs (**H**), showing the 12 and 13 largest categories.

Twelve individuals were included in this study: 6 patients with LAMA2-CMD and 6 age-matched unaffected controls. The University of Iowa collected all patient muscles under their IRB-approved protocol and provided specimens for research as deidentified muscle sections on glass slides. All biopsies were collected for initial diagnostic purposes. Due to limited availability, control tissue was age matched as closely as possible, and immunofluorescence (IF) was used to confirm the absence of laminin-α2 (Figure 1, A and B). Four out of 6 of the patients exhibited full laminin-211 deficiency (Figure 1, C and D), and 2 patients experienced partial laminin-α2 loss (Figure 1, E–H). Patient demographics and *LAMA2* variants are listed in Table 1. Two different types of ROIs were selected from each patient tissue biopsy: a small representative area and a single skeletal muscle fiber that includes muscle fibers and the surrounding microenvironment (Figure 2, A and B). In patient samples, area ROIs were selected from regions showing minimal muscle damage and immune cell infiltration based on morphological markers for desmin, CD68, CD31, and DNA (Figure 2A). To investigate potential muscle-specific protein changes, single-fiber ROIs were also included in this study (Figure 2B). Hypertrophic fibers and low-desmin-expressing fibers were avoided. Using NanoString’s GeoMx IO Proteome Atlas (IPA), 575 different proteins were analyzed (Supplemental Table 1; supplemental material available online with this article; https://doi.org/10.1172/jci.insight.194581DS1). After normalizing protein probe counts to negative controls (rabbit IgG, rat IgG2a, and rat IgG2b), heatmaps were generated through the Nanostring Data Analysis suite. Further downstream analysis in which volcano plots were generated identified differentially expressed proteins (DEPs), displaying 216 DEPs in the area ROIs and 40 DEPs in the single-fiber ROIs from the GeoMx IPA (Figure 2, C and D, and Supplemental Table 2). DEP analysis of the area ROIs revealed that out of the 216 DEPs, only 2 proteins were enriched in the unaffected group, and 214 were enriched in the patients with LAMA2-CMD (Figure 2E). The single-fiber ROI volcano plots displayed only 1 upregulated DEP in the unaffected group compared with 39 proteins in the LAMA2-CMD muscle fibers (Figure 2F). LAMA2-CMD DEPs from both ROI groups were then categorized using NanoString’s target group classification from the data analysis suite (Figure 2, G and H). Similar biological processes were observed in both area and single-fiber ROI data sets, including increased expression of proteins involved in the MAPK and PI3K/AKT pathways. Notably, the epithelial-mesenchymal transition (EMT) pathway had the highest number of DEPs in the single-fiber group. EMT results from transcription factors that alter gene expression to promote loss of cell-cell adhesion (19), suggesting a shift in cytoskeletal dynamics within the single fibers of patients with LAMA2-CMD (Figure 2H). Other protein biological process groups were associated with senescence, oxidative stress, and apoptosis. Furthermore, there was a large presence of DEPs associated with immune response activation, such as chemokine, interleukin, and TLR signaling (Figure 2, G and H).

### Age-dependent proteomic changes highlight progressive pathology in LAMA2-CMD muscle.

**Figure 3:**
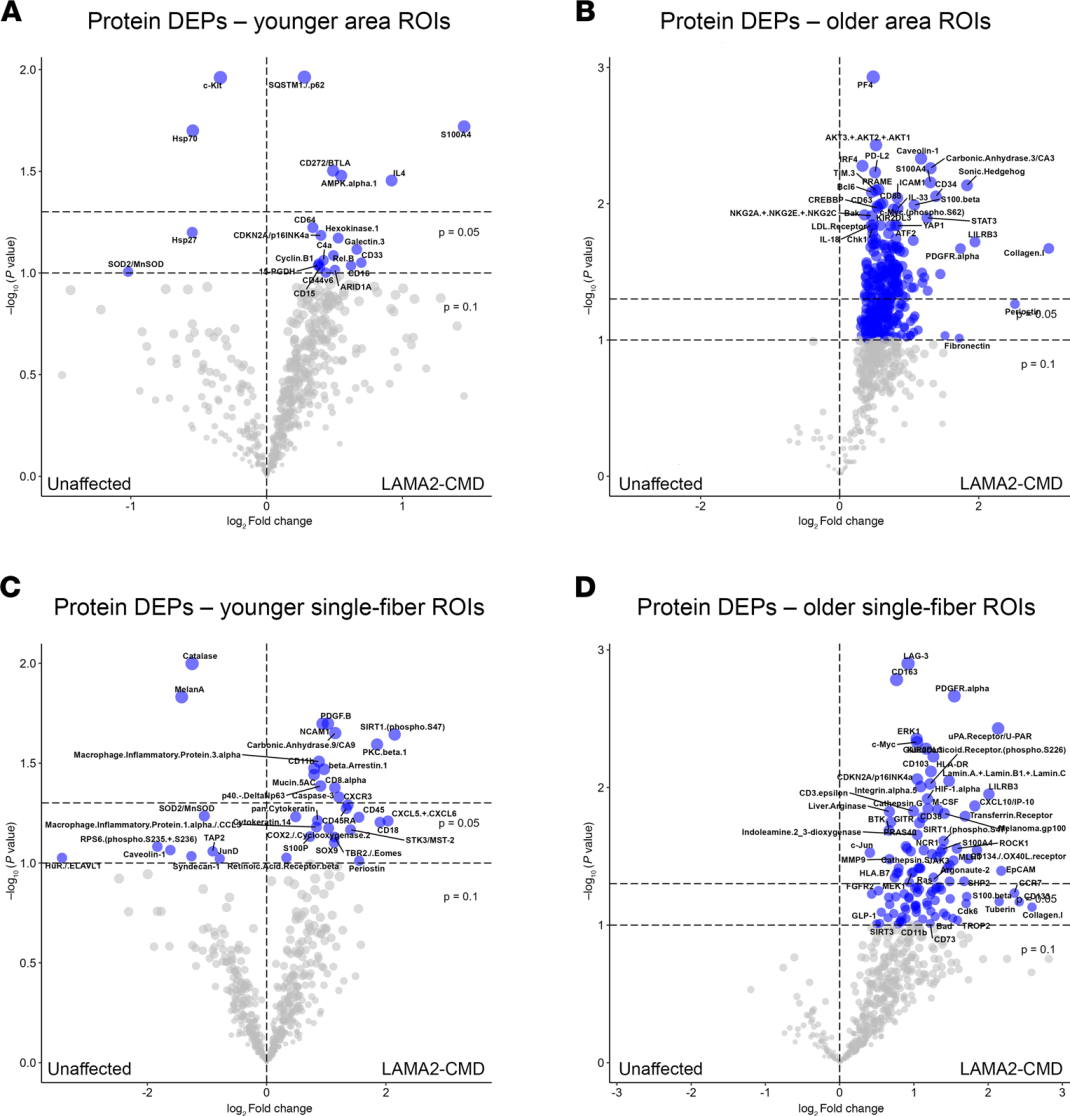
Age-sorted protein expression data reveal protein accumulation in older patients with LAMA2-CMD. (**A** and **C**) Volcano plots showing differentially expressed proteins (DEPs) in area and single-fiber ROIs from the younger cohort, comparing unaffected and LAMA2-CMD tissues. (**B** and **D**) Volcano plots showing DEPs in area and single-fiber ROIs from the older cohort, comparing unaffected and LAMA2-CMD tissues, illustrating protein accumulation over time in the LAMA2-CMD skeletal muscle microenvironment and single fibers.

For many patients with LAMA2-CMD, early symptoms can appear within the first week of life (20). Additionally, LAMA2-CMD symptoms, such as respiratory involvement and joint contractures, become more severe after the onset of adolescence (20). Therefore, we divided the dataset into younger and older patients, and their age-matched unaffected controls (Table 1). In the unaffected group, 4 DEPs were identified, while 18 enriched DEPs were found in the LAMA2-CMD younger patient ROIs (Figure 3A and Supplemental Tables 2 and 3). Notably, heat shock protein 70 (HSP70) and heat shock protein 27 (HSP27) were downregulated in the LAMA2-CMD group (Table 2). These molecular chaperones respond to stress stimuli, helping to maintain muscle fiber integrity and support muscle regeneration and recovery (21). The early loss of these proteins in patients with LAMA2-CMD may worsen skeletal muscle damage through protein accumulation and inhibit muscle regeneration over time.

In the older group, 322 DEPs were identified in the LAMA2-CMD area ROIs, while no enriched proteins were found in the unaffected group (Figure 3B). Collagen I showed the highest change, with a log_2_(fold change) (log_2_FC) of 3.0 (*P* = 0.021) in the older cohort ROIs but was not differentially expressed in the younger cohort, suggesting compensatory extracellular matrix (ECM) remodeling occurs over time (Figure 3, A and B). The single-fiber ROI datasets were also sorted by age, with younger and older patients compared to their age-matched controls (Figure 3, C and D). In younger patients, single myofibers displayed a mix of down- and upregulated DEPs (Figure 3C). Similar changes were also observed for the single-fiber ROIs compared with the area ROIs, primarily in the older group, where LAMA2-CMD skeletal muscle exhibited a disproportionate number of enriched DEPs — 113 in total — compared with zero DEPs in the unaffected group (Figure 3D). This large accumulation of proteins found in the muscle of older patients may be due to dysfunctional proteostasis due to high levels of cellular stress related to the severe muscle pathology, potentially due to the early loss of HSP70 and HSP27 (Figure 3a).

### Pathway analysis reveals age-dependent oxidative stress, apoptosis, and immune dysregulation in LAMA2-CMD muscle tissue.

**Figure 4:**
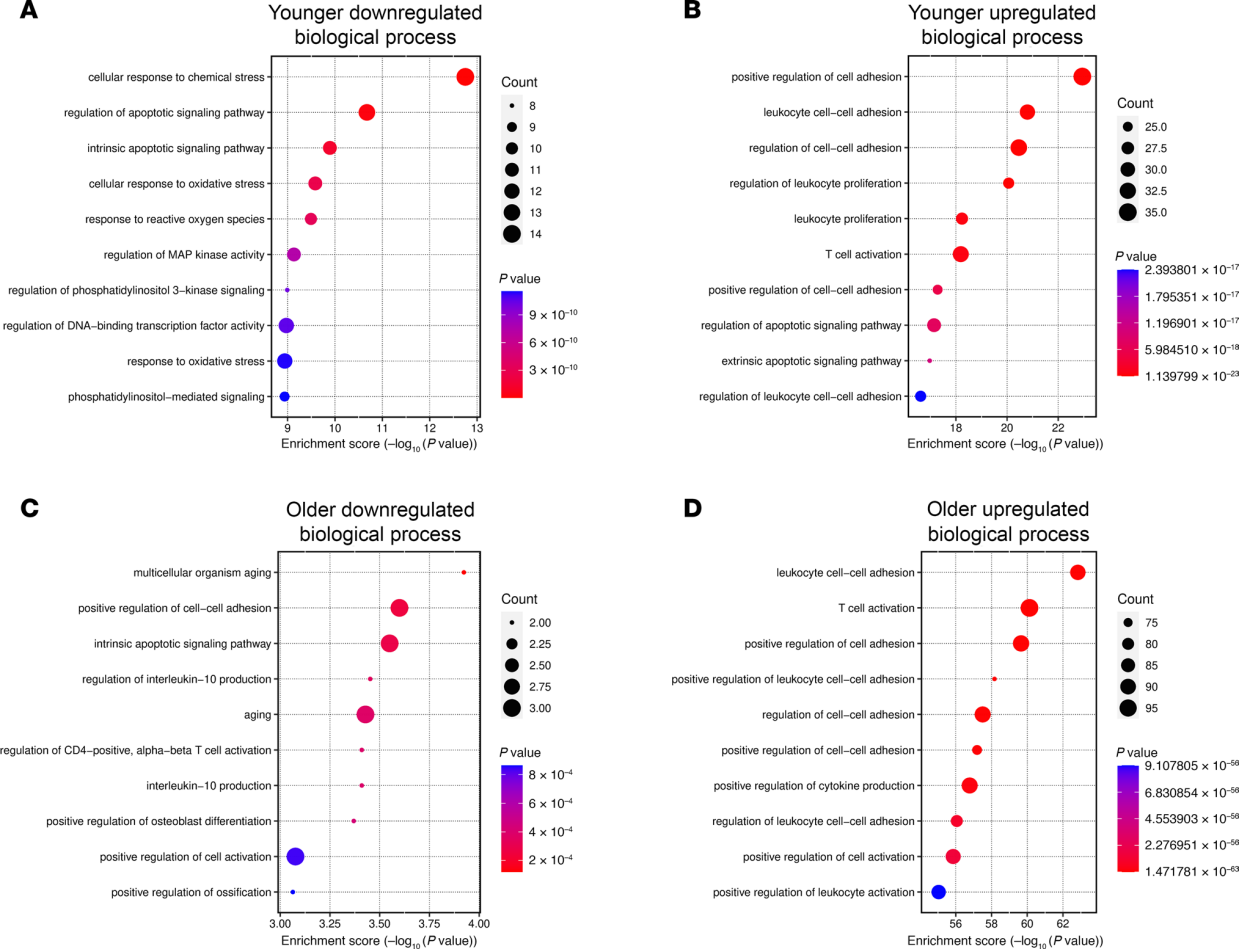
GO pathway analysis reveals altered biological processes as disease progresses in patients with LAMA2-CMD. (**A** and **B**) GO enrichment analyses of proteins with log_2_FC >0.25 for younger area ROI protein expression using down- and upregulated proteins, respectively. The 10 most down or upregulated biological processes are shown. (**C** and **D**) GO enrichment analyses of older area ROIs.

Gene Ontology (GO) pathway analysis was performed on DEPs from the age-sorted data area ROIs with a log_2_FC cutoff of 0.25 (Figure 4, A–D). Analysis of downregulated proteins in the younger area ROIs revealed changes in LAMA2-CMD patient response to chemical and oxidative stress, apoptosis, and reactive oxygen species (ROS), among other biological processes (Figure 4A). In the single-fiber ROIs, both younger and older patients also showed an upregulation in ROS response and metabolic processes (Supplemental Figure 1). This may be linked to the early downregulation of key enzymes, superoxide dismutase 2 (SOD2) (log_2_FC = –1.04, *P* = 0.06) in the muscle fiber and skeletal muscle area, as well as catalase (log_2_FC = –1.25, *P* = 0.01) in the single-fiber ROIs, which are enzymatically responsible for breaking down superoxide and hydrogen peroxide, respectively (22) (Table 2). Analysis of upregulated proteins shows increased activation in inflammatory and adhesion mechanisms, as well as T cell activation. Notably, extrinsic apoptotic signaling pathway was enriched, suggesting LAMA2-CMD patient skeletal muscle fibers may prefer extrinsic apoptosis over intrinsic apoptosis (Figure 4B). GO analysis of the older muscle area ROIs had some similarities to the younger muscle area ROI expression profiles, such as downregulation of intrinsic apoptosis and upregulation of leukocyte modulation, T cell activation, and cell-to-cell adhesion regulation (Figure 4, C and D). Older patient downregulated biological processes were also involved in the regulation of skeletal muscle aging and activation of helper T cells (Figure 2C).

### DSP analysis of dy^W–/–^ mouse skeletal muscle highlights metabolic and immune dysregulation similar to human LAMA2-CMD.

**Figure 5:**
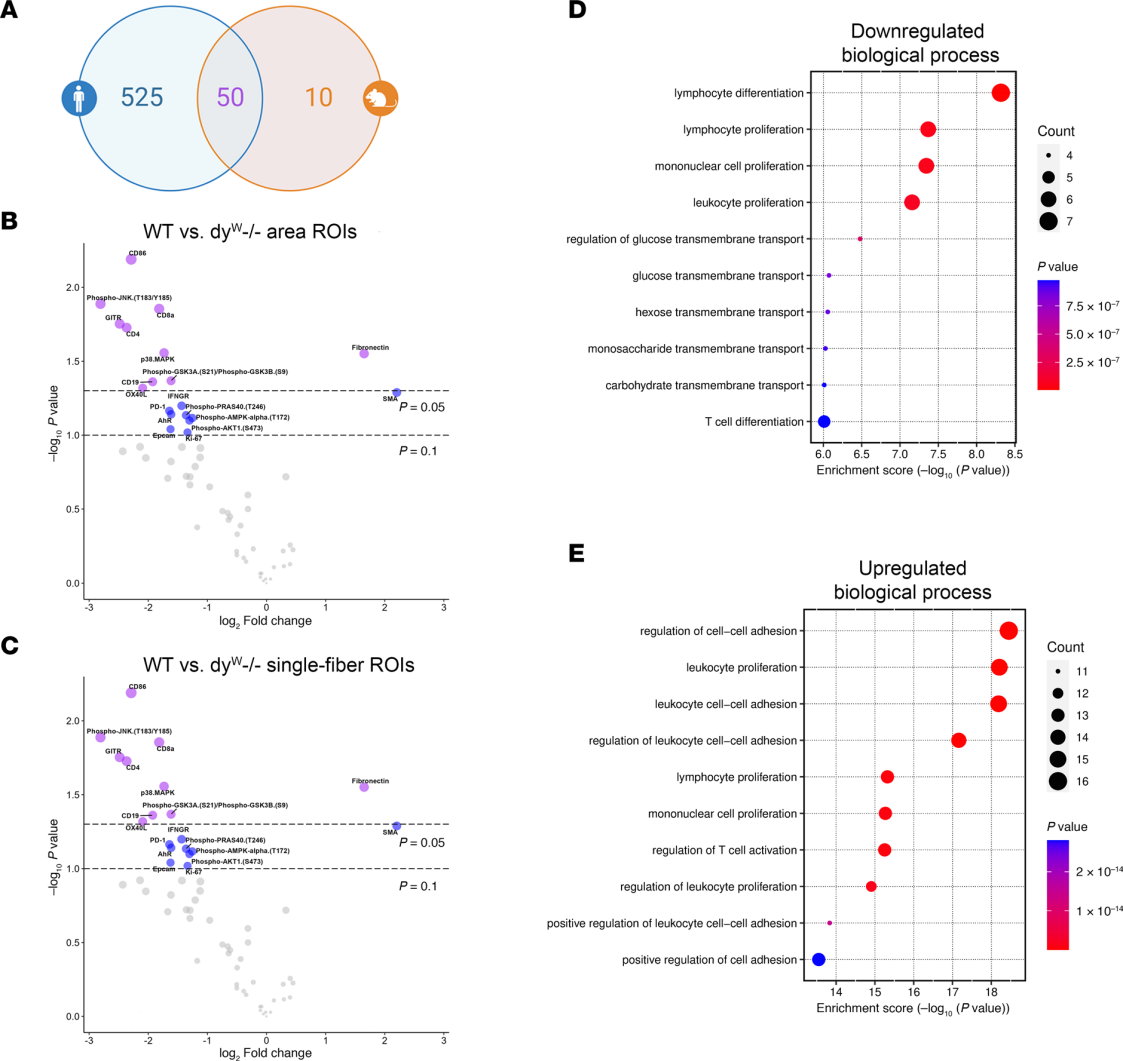
Digital spatial profiling of *dy^W–/–^* TA shows disrupted glucose metabolism and upregulated inflammatory processes. (**A**) Number of overlapping protein probes found in NanoString’s GeoMx human IPA panel and mouse immune-oncology panel. (**B** and **C**) Area and single-fiber ROI DEP volcano plots for WT compared to *dy^W–/–^*. (**D** and **E**) GO pathway analyses of proteins with log_2_FC >0.25 of down- and upregulated proteins. The 10 most down or upregulated biological processes are shown.

Using NanoString’s GeoMx Immuno-Oncology mouse panel, we performed DSP on wild-type (WT) and *dy^W–/–^* tibialis anterior (TA). The only commercially available mouse panel was much smaller than the human IPA panel. However, 50 of the 60 probes overlap with those in the human IPA panel (Figure 5A). ROIs were selected similarly to the human DSP, and the 60 different proteins were analyzed within the specified ROIs. Protein counts were normalized using negative controls (rabbit IgG, rat IgG2a, and rat IgG2b), and volcano plots were generated displaying DEPs of the area and single-fiber ROIs (Figure 5, B and C, and Supplemental Table 5). GO pathway analysis was performed on the downregulated and upregulated proteins between the *dy^W–/–^* and WT area ROIs (Figure 5, D and E). The downregulated biological processes indicate a dysregulation in lymphocyte development, as well as skeletal muscle metabolism. The downregulated proteins in the *dy^W–/–^* mouse involved in skeletal muscle metabolism include the biological processes of glucose, hexose, monosaccharide, and carbohydrate transmembrane transport (Figure 5D). Although the mouse protein panel is smaller than the human IPA panel, the upregulated biological processes matched 6 out of 10 biological processes in the younger patient area ROIs and shared 5 out of 10 upregulated biological processes found in the older patient area ROIs (Figure 5E). These upregulated biological processes include positive regulation of cell adhesion, leukocyte cell-to-cell adhesion, and T cell activation (Figure 4, B and D, and Figure 5E).

### RhLAM-111 treatment restores laminin adhesion complex protein localization in dy^W–/–^ mice.

**Figure 6:**
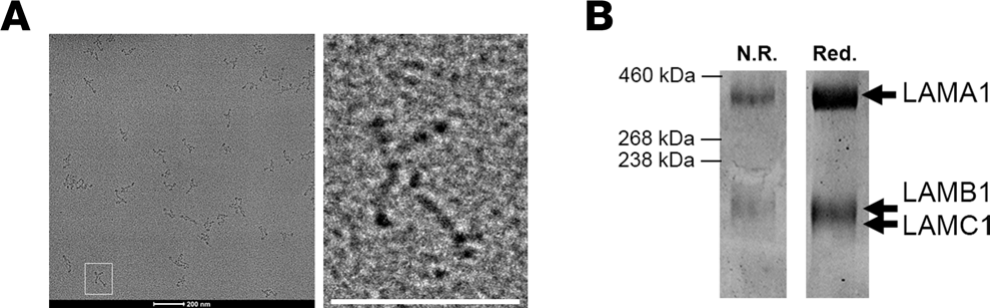
Characterization of purified rhLAM-111. (**A**) Shadow rotary image of purified rhLAM-111. Scale bar: 200 nm. (**B**) Coomassie stain of rhLAM-111 run in 4%–20% SDS-PAGE gel, illustrating purification of therapeutic and verification of laminin chain sizes.

RhLAM-111 was produced and purified using proprietary techniques by Sarcomatrix and Catalent with codon-optimized laminin α, β, and γ cDNA chains. Quality control was performed by the Burkin lab. Shadow rotary imaging showed the rhLAM-111 had a cruciform structure, which has been previously demonstrated for the laminin-111 structure (23) (Figure 6A). Using gel electrophoresis and Coomassie staining techniques, we confirmed rhLAM-111 protein purity and individual chains were the correct sizes (Figure 6B).

**Figure 7:**
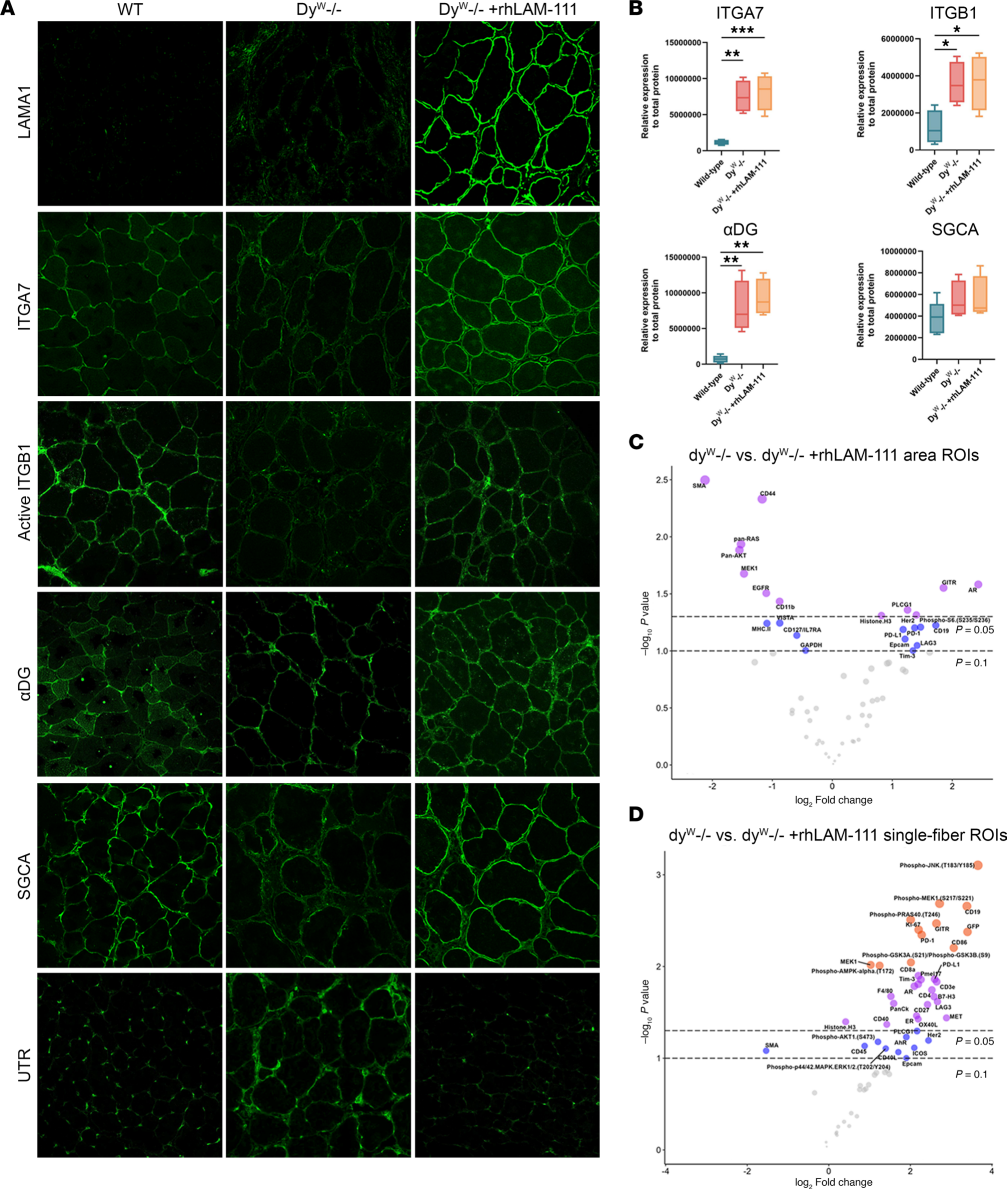
RhLAM-111 restores laminin adhesion complex proteins’ localization and restores protein expression profile to WT levels. (**A**) Localization of protein components of laminin adhesion complexes in the *dy^W–/–^* muscle. Transverse sections of whole TA muscle from 5-week-old WT, *dy^W–/–^* and *dy^W–/–^* after 7 days rhLAM-111 treatment with antibodies against LAMA1, ITGA7, ITGB1, α-dystroglycan (αDG), α-sarcoglycan (SGCA), and utrophin (UTRN). Antibodies were visualized using indirect fluorescence microscopy. Scale bar: 50 μm. (**B**) Western blot analysis was performed to measure and quantify ITGA7, ITGB1, αDG, and SGCA (relative to total protein). For Western blotting, 40 μg of protein was loaded per well. *n* = 5 samples for WT and *n* = 4 for *dy^W–/–^* and *dy^W–/–^* + rhLAM-111. (**C** and **D**) Area and single-fiber ROI DEP volcano plots for *dy^W–/–^* compared to rhLAM-111–treated *dy^W–/–^*. Data are shown as mean ± SD. *P < 0.05; **P < 0.01; ***P < 0.001 by 1-way ANOVA followed by Bonferonni post-hoc comparison between all groups.

We have previously demonstrated that treatment with mouse or human laminin-111 prevents disease progression and improves viability in the LAMA2-CMD mouse model, the *dy^W–/–^* mouse (12, 24). Integrin-α7 (ITGA7) deficiency, a characteristic prevalent in patients with LAMA2-CMD and *dy^W–/–^* mice, led us to investigate the localization of protein components within laminin adhesion complexes. These include the dystrophin-glycoprotein complex (DGC) and α7β1-integrin. Using IF, we observed that, compared with WT tissue, laminin adhesion complex components such as ITGA7, α-dystroglycan (αDG), and α-sarcoglycan (SGCA) localization to the sarcolemma were reduced in *dy^W–/–^* mice (Figure 7A). In contrast, rhLAM-111 treatment restored these laminin adhesion complex proteins to the sarcolemma. This restoration was rapid and observed at 48 hours after treatment, with full restoration achieved within 7 days after treatment (Supplemental Figure 2). Using an antibody that specifically recognizes activated integrin-β1 (ITGB1), we found that *dy^W–/–^* muscle had little activated ITGB1 on the muscle fiber surface, which represents ECM engagement. RhLAM-111 treatment increased the presence of activated ITGB1 to nearly WT levels (Figure 7A). Interestingly, utrophin, which is typically localized at the myotendinous junction, was localized throughout the myofiber in *dy^W–/–^* muscle. After rhLAM-111 treatment, utrophin’s localization around myofibers was diminished, and the localization was restored to WT localization (Figure 7A). Together, these results suggest that rhLAM-111 can effectively restore the altered laminin adhesion complex localization and promote activation of ITGB1 in *dy^W–/–^* skeletal muscle.

Western blot analysis in gastrocnemius muscle revealed substantial increases in laminin-adhesion complex proteins in *dy^W–/–^* mice compared with WT levels, including ITGA7 (*P* = 0.0013), ITGB1 (*P* = 0.0477), and αDG (*P* = 0.0037) (Figure 7B). In the contralateral rhLAM-111–treated leg, ITGA7 (*P* = 0.0006), ITGB1 (*P* = 0.0435), and αDG (*P* = 0.0011) protein levels were also significantly increased compared with WT (Figure 7B). Levels of SGCA were slightly increased in both the *dy^W–/–^* and *dy^W–/–^* + rhLAM-111 groups (Figure 7B). However, these differences were not statistically significant. Protein quantification was normalized to total protein, since common housekeeping controls such as GAPDH and actin can be altered in laminin-deficient muscle due to their roles in metabolism and cytoskeleton, respectively. Higher abundance of laminin-adhesion complex proteins may indicate a compensatory increase in protein production due to the inability of these proteins to anchor themselves within the sarcolemma properly. Previous studies have also indicated that DGC protein content is not a quality metric compared with the proper translocation to the sarcolemma (25, 26).

### Restoration of signaling pathways and immune modulation in dy^W–/–^ skeletal muscle following rhLAM-111 treatment.

Laminin adhesion complexes play a crucial role in signal transduction of skeletal muscle. Although the DGC is a key mechanosensor in skeletal muscle, it also acts as a scaffold for signaling proteins (27). The transmembrane receptor α7β1-integrin is involved in cellular adhesion to the ECM and regulates numerous signaling pathways, including PI3K/AKT, MAPK, and TGF-β/SMAD pathways (28, 29). Given the disrupted localization of laminin adhesion complex components and downregulation of active ITGB1 in the *dy^W–/–^* mice, we hypothesized that these changes could lead to disruptions in major signaling pathways.

To explore these potential changes, we utilized NanoString’s GeoMx Immuno-Oncology protein panel to assess protein expression in the TA muscle of *dy^W–/–^* mice. Mice were intramuscularly treated at 4 weeks of age with 5 μg/kg bodyweight of rhLAM-111 in the left TA for 7 days, while the right leg served as a contralateral control and received an equal volume of phosphate-buffered saline (PBS). WT tissue was collected from littermates of the same age. Volcano plots were generated to compare the differences between *dy^W–/–^* and *dy^W–/–^* + rhLAM-111 area and single-fiber ROI DEPs (Figure 7, C and D).

In the area ROIs between the *dy^W–/–^* and *dy^W–/–^* + rhLAM-111 groups, there is a mixture of DEPs. Among the 12 DEPs enriched in the LAM group, 10 were also enriched in WT compared with *dy^W–/–^* (Figure 5B and Figure 7C). Two of the overlapping proteins, glucocorticoid-induced TNFR-related protein (GITR) and T cell immunoglobulin and mucin-domain containing-3 (TIM3), are involved in immune regulation. GITR enhances regulatory T cells, and TIM3 dampens T cell immune responses (30, 31). These results suggest that *dy^W–/–^* mice may experience modulation of the T cell immune response after rhLAM-111 treatment. Additionally, rhLAM-111 restores histone H3 protein levels in *dy^W–/–^* mice. In the human IPA panel, 13 different histone H3 targets display various posttranslational modifications, with 9 histone H3 markers having an FC decrease of over 20% (Supplemental Table 6). The downregulation of histone H3 in patients and *dy^W–/–^* mice may indicate that epigenetic dysregulation could be a hallmark of LAMA2-CMD pathology.

In the single-fiber ROIs, rhLAM-111 treatments appear to target both the MAPK and PI3K/AKT pathways. The most significant protein expression change observed was a prominent increase in phosphorylated c-Jun N-terminal kinase (p-JNK), with a log_2_FC of 3.66 (*P* = 0.00078) (Figure 7D). p-JNK acts as a molecular switch that, when activated, stimulates muscle fiber growth, primarily influencing fast-twitch fibers (32). Given JNK’s key role in the MAPK signaling network, we examined the expression of other MAPK proteins and observed low levels of phospho-mitogen-activated protein kinase kinase 1 (p-MEK1), phospho-extracellular signal–regulated kinases 1/2 (p-ERK1/2), and serine/threonine-protein kinase B-raf (p-BRAF) in *dy^W–/–^* in comparison with WT, in which rhLAM-111 treatment was able to increase and restore these proteins in the contralateral leg. RhLAM-111 treatment also increased proteins associated with the PI3K/AKT signaling network, including p-AKT1 at Ser473 (log_2_FC = 1.21, *P* = 0.066), phospho-glycogen synthase kinase 3A/B (p-GSKA/GSK3B) (log_2_FC = 2.01, *P* = 0.0090), and phospho-proline-rich AKT substrate 40 kDa (p-PRAS40) (log_2_FC = 2.01, *P* = 0.0031) (Figure 7D). p-AKT1 at Ser473 is required for the activation of the PI3K/AKT signaling pathway. p-AKT1 also promotes the activation of mTORC1 by facilitating the phosphorylation of downstream effectors such as PRAS40 (33). Another downstream target of AKT, p-GSK3A/GSK3B, is essential for glycogen metabolism and contributes to an anabolic state in skeletal muscle (34). However, there was also a significant increase in phospho-protein kinase, AMP-activated, α1 catalytic subunit (p-PRKAA1) (log_2_FC = 1.25, *P* = 0.0098), which initiates catabolic processes in skeletal muscle to generate ATP (35) (Figure 7D).

When comparing WT to rhLAM-111–treated *dy^W–/–^* single-fiber ROIs, WT expressed no enriched proteins, whereas rhLAM-111–treated samples showed 3 (Supplemental Figure 3). These findings suggest that rhLAM-111 treatment restores protein expression patterns similar to WT tissue after 7 days. The only enriched DEP between rhLAM-111 and WT was fibronectin (log_2_FC = 2.34, *P* = 0.033), which may be a result of the short-term treatment. Fibronectin levels may further be reduced with longer-term treatment.

### RhLAM-111 treatment mitigates metabolic dysfunction and restores protein trafficking in early-stage LAMA2-CMD.

*Dy^W–/–^* mice were treated at 4 weeks of age and euthanized at 5 weeks, an age range chosen to better represent the younger cohort of patients in terms of disease progression and pathology. Consequently, we focused on protein expression levels and FCs in this younger cohort. The top 3 downregulated proteins identified in the younger ROI areas compared with the unaffected group were glucose transporter protein type 1 (GLUT1), ELAV-like RNA-binding protein 1 (ELAVL1), and insulin-like growth factor 1 receptor (IGF1R), all of which are critical to glucose metabolism and substrate availability in skeletal muscle (Table 2).

**Figure 8:**
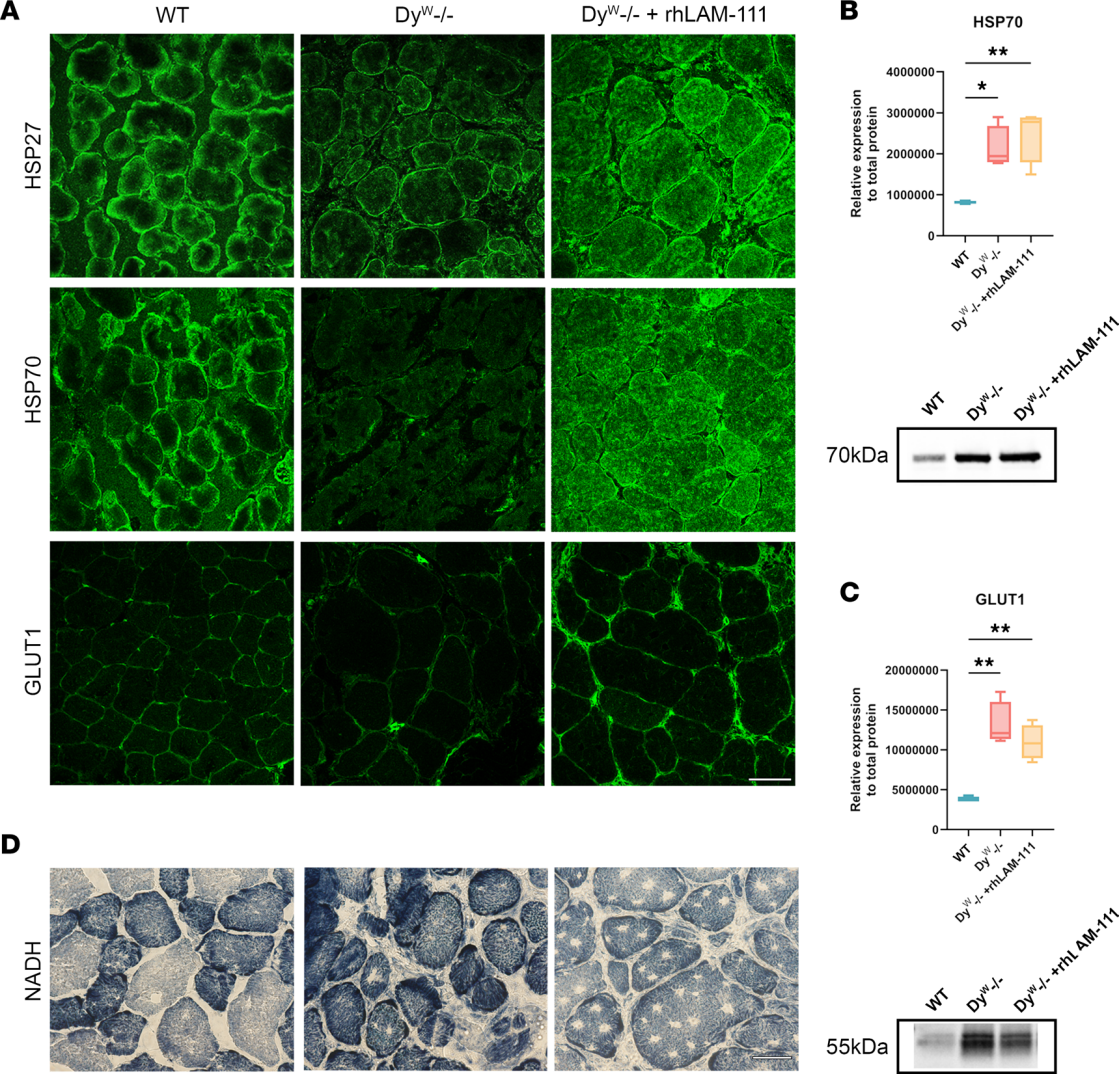
RhLAM-111 treatment increases cytosolic heat shock protein expression and enhances GLUT1 localization to the sarcolemma after 7 days of treatment. (**A**) IF imaging of HSP27, HSP70, and GLUT1 in WT, *dy^W–/–^*, and *dy^W–/–^* + rhLAM-111 (contralateral legs). HSP27 and HSP70 are localized to skeletal muscle cytoplasm, which is associated with protein chaperone and unfolding activity. GLUT1 expression on the muscle fiber sarcolemma increases with rhLAM-111 treatments. Scale bar: 50 μm. (**B**) Western blot analysis was performed to measure and quantify HSP70 (relative to total protein). *n* = 4 per group. (**C**) Western blot quantification of GLUT1 (relative to total protein). *n* = 3 samples for WT and *n =* 4 for *dy^W–/–^* and *dy^W–/–^* + rhLAM-111 groups. (**D**) Enzyme histochemistry for NADH showing high levels of accumulation in PBS-treated *dy^W–/–^* TA compared with WT. RhLAM-111 restores NADH expression similar to WT levels in 7 days after treatment. Data are shown as mean ± SD. *P < 0.05; **P < 0.01 by 1-way ANOVA followed by Bonferonni post-hoc comparison between all groups.

HSP27 and HSP70 showed reduced expression in the younger patients. IF was performed to look at the proteins’ expression patterns in the *dy^W–/–^* muscle and to explore laminin’s potential role. In *dy^W–/–^* TA muscle, HSP27 was localized to the membrane and was largely absent from the cytosol (Figure 8A). Following rhLAM-111 treatment, HSP27 exhibited cytosolic localization. HSP70 showed a similar pattern, with rhLAM-111 treatment inducing a substantial shift toward cytosolic localization (Figure 8A). The disrupted localization of HSP27 and HSP70 in laminin-α2–deficient muscle, combined with the disproportionately increased protein enrichment observed in older patients, highlights laminin’s critical role in regulating myofiber protein trafficking and expression.

GLUT1 localization was investigated using IF in WT and *dy^W–/–^* TA muscle. In *dy^W–/–^*, there was an absence of sarcolemmal GLUT1 localization (Figure 8A). However, in the rhLAM-111–treated contralateral leg, GLUT1 localization was restored to the sarcolemma. GLUT1 distribution also became more uniformly distributed around muscle fibers. These findings may suggest why there is a higher proportion of oxidative fiber types in LAMA2-CMD patient muscle, further elucidating a role for laminin in regulating metabolic function in muscle.

Protein levels of HSP70 and GLUT1 were significantly elevated in both *dy^W–/–^* and *dy^W–/–^* + rhLAM-111 skeletal muscle (Figure 8, B and C), a finding that contrasts with the human spatial proteomics data (Figure 2A and Table 2). HSP70 is a highly dynamic protein, and the molecular chaperone’s localization is critical for its biological activity (36). HSP70 is also highly expressed in macrophages and other inflammatory cells that are found in high abundance within the interstitial space of *dy^W–/–^* myofibers (37, 38). Western blot analysis was performed on whole muscle tissue lysate, whereas the patient’s spatial proteomic ROIs consist of single fibers and muscle fiber areas with low immune cell infiltrate and intact muscle fibers. Similarly, the elevated GLUT1 signal may reflect contributions from proliferative myofibroblasts, which rely heavily on aerobic glycolysis (39, 40). Inflammatory cells such as neutrophils and macrophages also express profuse levels of GLUT1 due to high metabolic demands (41–43). Altogether, these findings underscore that protein localization, rather than absolute abundance, provides the more biological relevance behind the pathomechanisms of dystrophic tissue.

GO pathway analysis of patient DEPs, as well as previous proteomic studies, demonstrated that LAMA2-CMD muscle has bioenergetic imbalances that contribute to constant oxidative stress and ROS accumulation (44). To assess metabolic activity, nicotinamide adenine dinucleotide (NADH) precipitate enzyme histochemistry was performed. Compared with WT, *dy^W–/–^* myofibers appeared much darker, and NADH accumulation was found throughout the entire fiber (Figure 8D). Within 7 days of rhLAM-111 treatment, NADH accumulation in the contralateral leg was reduced to levels comparable to WT. High levels of NADH do not directly indicate high levels of ROS, but rather the higher levels of NADH are indicative of impaired oxidative phosphorylation (OXPHOS) and therefore causes ROS accumulation. High NADH levels suggest a bottleneck in the mitochondrial electron transport chain, specifically at complex I, where NADH donates electrons (45).

### Downregulated spatial omics proteins, HSP27, HSP70, and GLUT1, display altered localization in patients with LAMA2-CMD.

**Figure 9:**
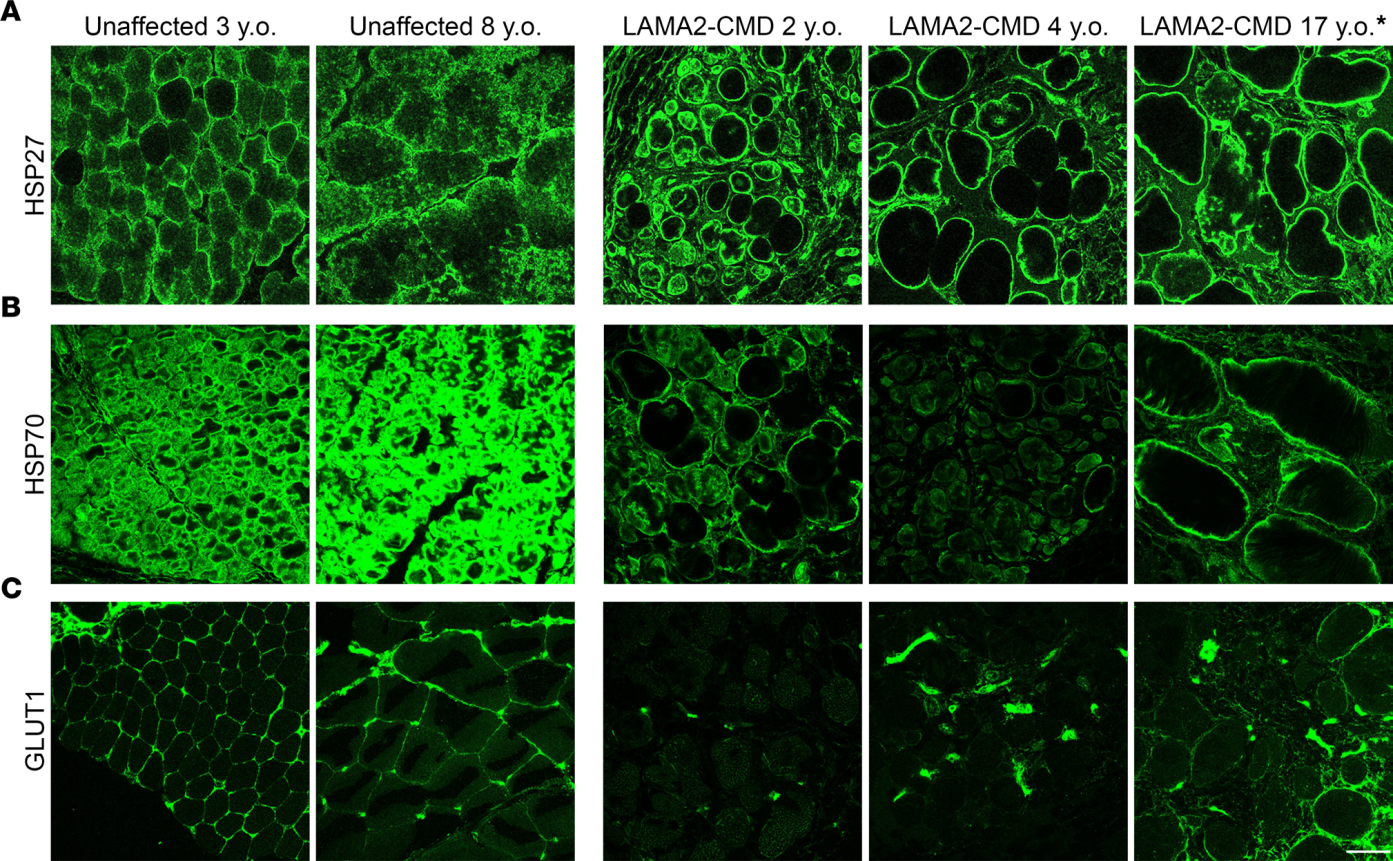
Patients with LAMA2-CMD exhibit altered localization of downregulated DSP proteins HSP27, HSP70, and GLUT1. (**A**–**C**) IF imaging of HSP27, HSP70, and GLUT1 in unaffected and LAMA2-CMD muscle biopsies. *The 17-y.o. patient has a partial laminin deficiency. Scale bar: 50 μm.

HSP27 and HSP70 are highly dynamic molecular chaperone proteins that localize to different areas of skeletal muscle fibers in response to various stimuli (46). In unaffected patients, HSP27 can be found dispersed throughout the cytoplasm, where it binds to the cytoskeleton to help maintain structural integrity (Figure 9A). In response to mechanical stress or injury, HSP27 will rapidly translocate to the cell membrane, particularly near the Z-disks. In LAMA2-CMD, HSP27 can be seen heavily aggregated around the sarcolemma across varying ages and disease states (Figure 9A). Like HSP27, HSP70 is located intracellularly in the cytoplasm of myofibers; however, in response to injury and stress, HSP70 relocates to the sarcolemma (36, 46). By binding to the damaged proteins near the sarcolemma, HSP70 prevents them from aggregating and facilitates their repair. In unaffected tissue, HSP70 could be found heavily localized within the cytoplasm of myofibers (Figure 9B). However, similarly to HSP27, HSP70 was concentrated primarily around the myofibers of the 2-year-old (2-y.o.) and 17-y.o. patients (Figure 6B). In the 4-y.o. LAMA2-CMD patient, HSP70 IF staining appeared to be overall reduced and could be correlated with disease progression in patients with full laminin deficiency.

GLUT1 localization was also investigated using IF in the patient tissues. In the unaffected controls, GLUT1 could be found uniformly distributed around the myofibers (Figure 9C). However, GLUT1 staining was almost completely diminished in the 2-y.o. and 4-y.o. LAMA2-CMD patients. Partial GLUT1 staining could be seen in the 17-y.o. patient tissue. This partial staining could potentially be correlated with the patient’s partial laminin deficiency, and GLUT1 could be localized to the sarcolemma in regions where the laminin is present (Figure 9C).

### Cytosolic p-JNK localization decreases alongside disease progression in LAMA2-CMD muscle.

**Figure 10:**
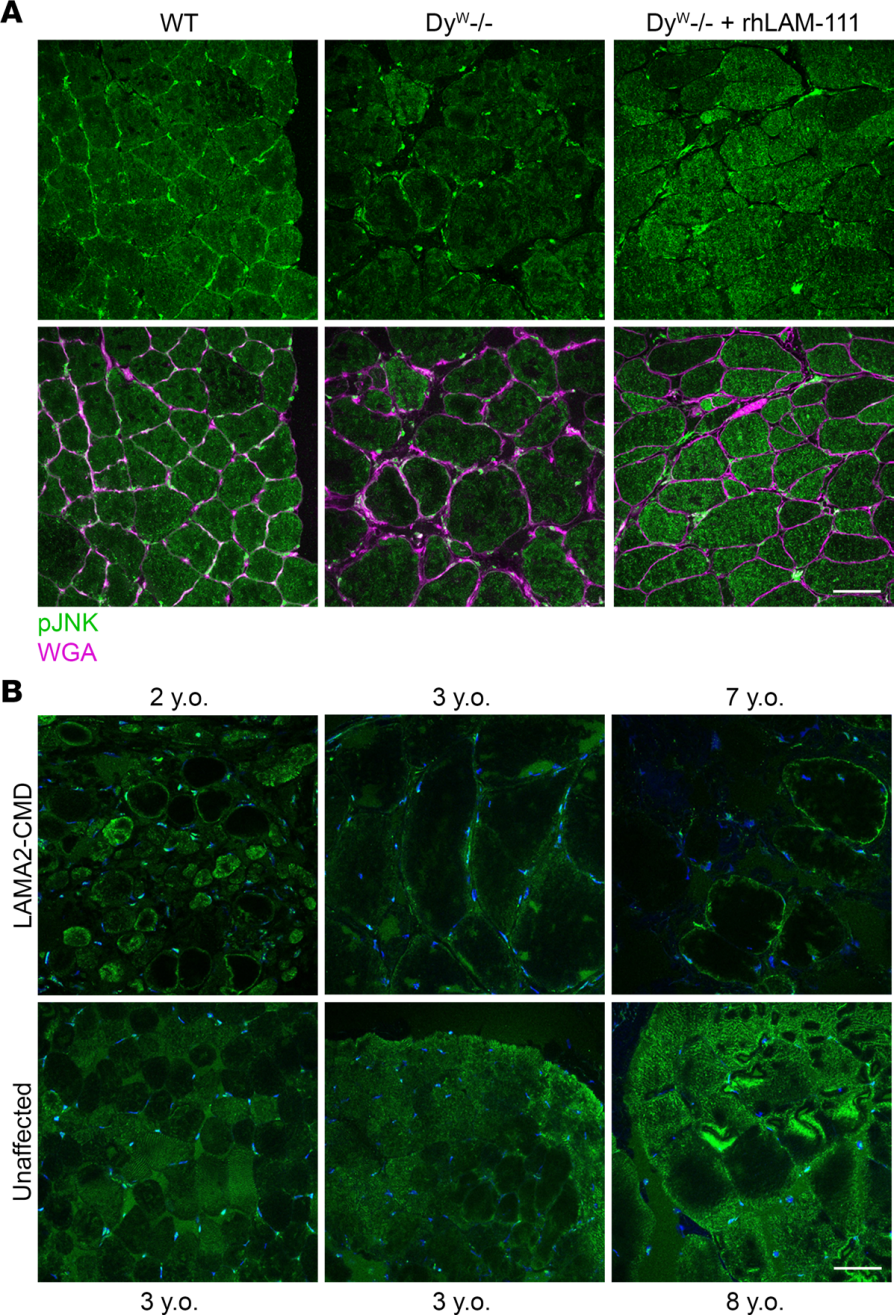
Cytosolic localization of p-JNK in *dy^W–/–^* and LAMA2-CMD skeletal muscle. (**A**) IF of p-JNK in WT, *dy^W–/–^*, and *dy^W–/–^* + rhLAM-111. p-JNK localization in the cytoplasm is decreased in the *dy^W–/–^* muscle and rhLAM-111 treatments increase cytosolic p-JNK after 7 days. Images on the bottom row are with wheat germ agglutinin (magenta) to highlight the muscle fiber sarcolemma. Scale bar: 50 μm. (**B**) IF of p-JNK in patients with LAMA2-CMD and age-matched controls show a decreasing presence of p-JNK in the cytosol as patients age. Scale bar: 50 μm.

Since p-JNK exhibited the largest FC with high significance, IF was performed to observe the localization in mice (Figure 10A). Higher levels of p-JNK were localized in the cytosol of WT and *dy^W–/–^* + rhLAM-111 mouse myofibers compared with *dy^W–/–^*. In human studies, no significant changes in JNK1/JNK2/JNK3 expression was detected in younger patients. However, the IPA panel used in these studies measured only total JNK, not the activated phosphorylated form of JNK used in the mouse panel.

Next, IF was utilized to observe the localization of p-JNK in patients with LAMA2-CMD. LAMA2-CMD patients ages 2, 3, and 7 y.o. were included in the panels, with unaffected controls 3, 3, and 8 y.o. (Figure 10B). In the 2-y.o. patient, p-JNK was present in the cytosol in some muscle fibers; however, in the 3-y.o. patient, p-JNK began to migrate to the sarcolemma of the myofibers, with some p-JNK present in the peripheral cytoplasm. The 7-y.o. patient’s cytosolic p-JNK was almost completely diminished, and some fibers had p-JNK strongly localized to the sarcolemma. Meanwhile, in the unaffected control tissue, p-JNK was present in the cytosol in most fibers, suggesting cytosolic p-JNK may be present in a preferred muscle fiber type.

## Discussion

In this study, we used spatial proteomics combined with comprehensive pathway analyses to demonstrate that profound changes in laminin-binding adhesion-signaling complexes play a crucial role in disease progression in patients with LAMA2-CMD and the *dy^W–/–^* mouse model. These findings contribute to our understanding of how the loss of crosstalk between laminin and its adhesion complexes contributes to disease pathology and highlight potential targets for therapeutic intervention.

**Figure 11:**
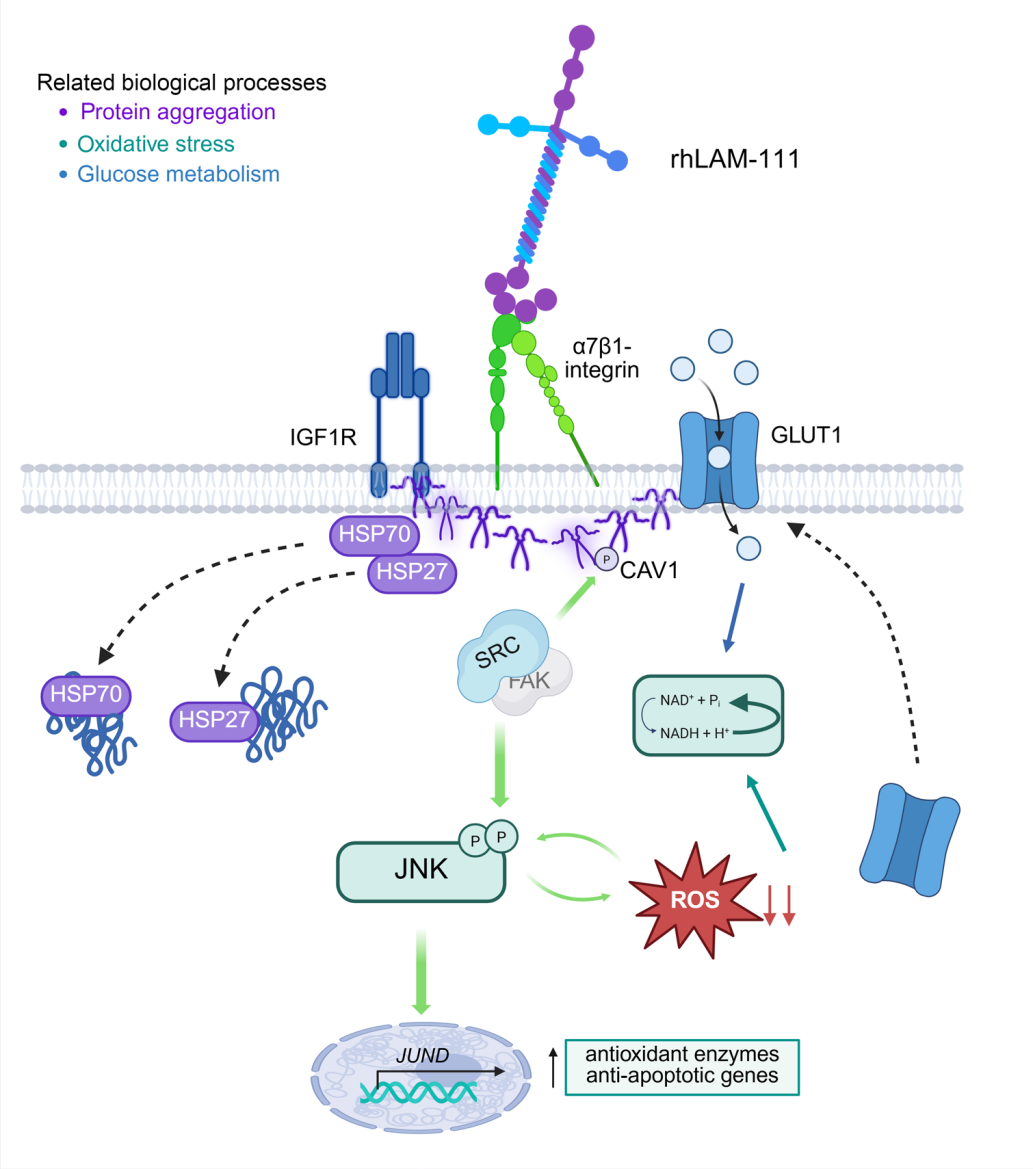
Schematic of molecular interactions between dysregulated spatial proteomics proteins’ biological processes and related pathways. All proteins included in the schematic are disrupted in human or mouse spatial proteomics and IF. Molecular interactions and associations are assumed based on peer-reviewed literature.

Using data from spatial proteomics and IF, we propose a mechanism of action by which laminin alleviates ROS accumulation through α7β1-integrin (Figure 11). Patients with LAMA2-CMD can experience secondary α7β1-integrin, and in the absence of laminin, α7β1-integrin remains in an inactive closed state and is not properly localized to the sarcolemma, preventing downstream signaling (47–49). When rhLAM-111 enters the basal lamina, it binds to integrin-α7, which then activates integrin-β1 (Figure 7A). Integrin-β1 regulates several proteins, including caveolin-1, GLUT1, and IGF1R, all of which are found to be downregulated in patients with LAMA2-CMD (Table 2) (50–52). Upon activation, integrin-β1 activates the proto-oncogene tyrosine-protein kinase (Src), which directly phosphorylates caveolin-1; this activation influences caveolin-1’s stabilization and downstream signaling (53). Activating caveolin-1 facilitates the translocation of GLUT1 and IGF1R to the cell membrane, enabling glucose influx and adaptation to hypoxic conditions. Restoration of the laminin adhesion complexes also promotes proper mechanotransduction signaling via JNK, which is directly phosphorylated and activated by Src. This in turn enhances c-Jun transcription, promoting glycolytic activity and enhancing GLUT1 expression (54–57). GLUT1 plays a key role in regulating ROS, unlike GLUT4, which has no such association (58). Activated JNK undergoes continuous autophosphorylation in response to ROS recognition, while a metabolic switch from oxidative to glycolytic further reduces ROS accumulation (59, 60). This shift alleviates muscle stress signals, leading to the dispersion of protein accumulation or stress granules. RNA stabilization, mediated by the RNA-binding protein ELAVL1, is supported by the activity of chaperone proteins HSP27 and HSP70, which aid in the unraveling process within the cytosol (61, 62). The increase in cytosolic HSP27 and HSP70 inhibits the JNK-mediated route of apoptosis through binding to the adaptor molecule Daxx (63, 64).

Analysis of younger patients with LAMA2-CMD identified early downregulation of molecular chaperones HSP27 and HSP70, proteins critical for stress response and muscle regeneration (21) (Table 2). The loss of these chaperones likely exacerbates skeletal muscle damage and impairs regenerative properties, further advancing disease progression. The reduction in antioxidant enzymes, such as SOD2 and catalase, may heighten oxidative stress in younger patients, contributing to early muscle fiber damage and apoptosis. Older patients with LAMA2-CMD displayed elevated accumulation of many DEPs compared with healthy controls, suggesting problems with muscle fiber autophagy, a known issue for DMD (65) or regulation of proteostasis. Downregulation of proteins is critical for maintaining ROS, plus protein accumulation over time may indicate stress granule formation within the myocytes due to oxidative stress found in patients with LAMA2-CMD and *dy^W–/–^* mice (66, 67). Our studies revealed that activated JNK in LAMA2-CMD patient tissue became more deficient in the cytosol as patients age. Typically, elevated levels of ROS are associated with JNK activation, but in patients with LAMA2-CMD and *dy^W–/–^* mice, this feedback mechanism appears to be dysregulated (68). Since cytosolic p-JNK is restored through rhLAM-111 treatments, JNK signaling may be regulated or initiated through laminin adhesion complexes.

Previous proteomic and transcriptomic studies have similarly observed deficiencies in LAMA2-CMD muscle metabolism, resulting from the downregulation of numerous proteins involved in OXPHOS and glycolysis (44, 69–71). Furthermore, the use of antioxidants in the *dy^2J^*/*dy^2J^* LAMA2-CMD mouse model decreases inflammation and oxidative stress in skeletal muscle (72). Our spatial proteomics findings align with these previous studies, revealing deficiencies in glycolysis-related proteins, as well as key mitochondrial antioxidant enzymes SOD2 and catalase in the younger patient tissue. The 3 greatest FC reductions were in glycolysis-related proteins: GLUT1, ELAVL1, and IGF1R. GLUT1 facilitates glucose uptake in the cell and is a rate-limiting step in the process of glycolysis (43, 73). IGF1R also plays a marked role in glycolysis regulation and is further involved in muscle regeneration (74). Studies have shown that overexpression of a precursor for muscle-specific IGF-1 improved muscle regeneration and longevity in laminin-α2–deficient mice, highlighting the importance of restoring the glycolytic ability of laminin-deficient muscle (75). On the other hand, ELAVL1 is not directly involved in glycolysis, but plays a role in skeletal muscle metabolic flexibility and stabilizes mRNA of many important glycolytic proteins, including GLUT1 (76, 77). Reduced expression of these proteins likely leads to reduced myofiber glucose availability and slow-twitch fiber-type prevalence observed in patients with LAMA2-CMD (78). Altogether, these findings underscore how metabolic deficiencies in laminin-deficient muscle contribute to disease pathology and highlight metabolic restoration as a promising therapeutic.

In the *dy^W–/–^* mouse model, rhLAM-111 treatment demonstrated the ability to restore key components of the laminin adhesion complex, including ITGA7, active ITGB1, and αDG, to their normal cellular localization. Restoration of the localization of proteins is likely critical for proper signal transduction and structural integrity of the muscle. Western blot analysis revealed significant increases in the protein levels of ITGA7, ITGB1, and αDG in *dy^W–/–^* compared with WT mice. These proteins may not be properly localized to the sarcolemma without the presence of laminin, and therefore, myofibers may produce more proteins in order to compensate for the laminin adhesion complex proteins. Protein quantification of HSP70 and GLUT1 was also elevated in the *dy^W–/–^* mice, which could be associated with fibroblasts and immune cells found in the dystrophic tissue. Previous studies have demonstrated that the localization of the DGC is a better outcome measure than protein quantification (25, 26). Therefore, spatial omics studies using defined ROIs, in combination with IF analysis, can provide valuable insights into the molecular and cellular events occurring within dystrophic skeletal muscle.

RhLAM-111 treatment effectively restored MAPK and PI3K/AKT signaling pathways, which are both essential for muscle growth, apoptotic regulation, and metabolic balance. Increased p-JNK and p-AKT1 suggest that rhLAM-111 treatment promotes an anabolic state beneficial to muscle repair while maintaining the myofiber energy balance via the coactivation of catabolic p-PRKAA1 (32, 79, 80). The restoration of histone H3 protein levels also suggests that rhLAM-111 may play a role in epigenetic dysregulation, a potential hallmark of LAMA2-CMD pathology.

While our findings elucidated signaling pathway and metabolic dysregulation in laminin-α2–deficient muscle alongside the therapeutic effects of rhLAM-111, several limitations are acknowledged. First, our study included a relatively small cohort of patients with LAMA2-CMD and age-matched unaffected controls for DSP and should be expanded to validate our findings. The limited availability of patient tissue constrained the power of our analyses, leading us to instead focus on effect sizes and biological relevance. Second, the *dy^W–/–^* mice were treated with rhLAM-111 for only 7 days, which may represent only the initial DEP changes and limit our understanding of the benefits or limitations of long-term treatment. For example, Western blot analysis showed significantly higher amounts of protein in both *dy^W–/–^* groups compared with WT levels. However, 7 days may not be a sufficiently long treatment time to be able to observe any possible stabilization of protein levels with rhLAM-111 administration. Additionally, this short-term treatment window may not reveal any potential immune response that the *dy^W–/–^* mouse could develop from an adaptive immune response to a recombinant human protein. There is also always a possibility for an immune response to develop to a large dose of protein therapeutic in patients due to potential glycosylation changes between native and recombinant protein isoforms and innate immune activation. Third, the NanoString GeoMx panels used in this study contained a limited number of probes, particularly in the mouse panel, which limits our ability to perform comparisons to patients with LAMA2-CMD. Expanding the DSP panels to include additional protein targets could provide a more robust investigation of the similarities and differences between the LAMA2-CMD human and mouse model signaling pathways.

In conclusion, this study advances our understanding of LAMA2-CMD by elucidating the complex interplay of dysregulated proteostasis, oxidative stress, and metabolic deficiencies in both human patients and the *dy^W–/–^* mouse model. Using spatial proteomics and pathway analyses, we identified key downregulated DEPs in younger patients with LAMA2-CMD, including HSP27, HSP70, and caveolin-1, which all play a key role in protein trafficking, folding, and stabilization. Additionally, antioxidant enzymes SOD2 and catalase were also downregulated. Further studies in *dy^W–/–^* mice illustrated the dysregulation of the laminin adhesion complexes, which were later restored with rhLAM-111 treatments, including the activation of ITGB1. DSP mouse studies identified several rescued signaling proteins, including p-JNK, p-GSK3A/GSK3B, and p-PRKAA1, all of which play crucial roles in skeletal muscle metabolism. The improper localization of GLUT1 and pathway analysis in the human studies also indicated increased oxidative stress and decreased glycolysis. Our spatial proteomics findings align with previous transcriptomic and proteomic human and mouse studies, which have also described mitochondrial bioenergetic impairment and ROS vulnerability (44, 69, 71, 72, 81). Increased NADH accumulation has been observed in LAMA2-CMD patient tissue (63), prompting us to assess NADH levels in the *dy^W–/–^* mice using immunohistochemical tissue staining. *Dy^W–/–^* muscle also exhibited abnormal accumulation of NADH, which could be attenuated after 7 days of rhLAM-111 treatment in the contralateral leg.

These findings underscore the contribution of laminin-associated pathways to muscle disease progression and highlight new potential therapeutic targets for LAMA2-CMD. RhLAM-111’s therapeutic potential is highlighted through its ability to restore key adhesion-signaling complexes, such as the activation of α7β1-integrin, its influence over the localization of protective molecular chaperones, and its promotion of a more glycolytic state. These findings pave the way for future studies exploring the clinical application of rhLAM-111 and other laminin-based therapies to mitigate disease progression and improve outcomes in LAMA2-CMD.

## Methods

### Sex as a biological variable.

For this study, sex was not considered as a biological variable. LAMA2-CMD is a rare pediatric muscular dystrophy; therefore, tissue biopsy availability is extremely limited. We selected a mixture of both male and female human tissues for this study since LAMA2-CMD can affect both males and females. For mouse experiments, both males and females were used, and findings were similar for both sexes.

### RhLAM-111.

RhLAM-111 was a gift from Sarcomatrix Therapeutics Corporation, produced using a proprietary cell line and purification process. The rhLAM-111 used in this study was reported to have a 99.2% purity level, and the identity was verified using LC-MS and Western blot analysis during the production process. Prior to use, rhLAM-111 was thawed at 4°C overnight prior to mouse injections.

### Animal treatments.

The *dy^W–/–^* mice were maintained on the same genetic background as previously described (82). Mice were housed within the Center of Molecular Medicine animal facilities with a 12-hour light/12-hour dark cycle. The *dy^W–/–^* mice were produced from breeding heterozygous *dy^W+/–^* mice with littermates used as controls. Homozygous *dy^W–/–^* mice were injected via intramuscular injections with rhLAM-111 or PBS at 4 weeks of age. The *dy^W–/–^* mice were injected intramuscularly one time in the left TA at 5 μg/kg bodyweight of rhLAM-111. The contralateral right TA muscle served as a control and was injected with an equal volume of PBS. For Western blot analysis, the left gastrocnemius was treated with 5 μg/kg bodyweight of rhLAM-111 intramuscularly and the contralateral leg was injected with equal volume of PBS. WT littermates were used as controls.

### Patient and mouse muscle for DSP.

Unaffected and LAMA2-CMD patient flash-frozen tissue was sectioned at 5 μm thickness, adhered to Surgipath X-tra adhesive slides (Leica Biosystems, 3800200), and then immediately transferred to dry ice.

*Dy^W–/–^* and WT TA muscles were harvested 7 days after injection (5 weeks of age) and immediately frozen in a liquid nitrogen–cooled isopentane bath. Later, tissues were formalin-fixed, paraffin-embedded, and then sectioned at 5 μm thickness and mounted to adhesive slides following standard protocol by Premier Laboratory LLC. Prepared slides were then shipped to NanoString’s TAP lab).

### NanoString proteomics.

DSP IF images with morphological markers for DNA, desmin, CD31, and CD68, provided and stained by NanoString, were used to select ROIs. CD68 and CD31 were utilized as morphological markers to identify immune infiltration within the interstitial space between muscle fibers and were used to guide area ROI selection. Single-fiber and area ROIs were selected for each human and mouse tissue. Muscle regions with the least amount of muscle damage and elevated desmin expression levels were intentionally selected. Immunohistochemistry was performed on rhLAM-111–treated mouse TA using anti-LAMA1 (Aviva Systems Biology, OACD05111; 1:300) to confirm the presence of rhLAM-111 in the TA and guide ROI selection for the treatment group. Multiplex DSP of protein in fresh-frozen and paraffin-embedded tissues was performed by NanoString Technologies using their GeoMx IPA human protein panel and GeoMx Immuno-Oncology mouse protein panel as part of the Technology Access Program, as per Merritt et al. (18).

### IF.

TA muscles were harvested, embedded in optimum cutting temperature (Thermo Fisher Scientific), and flash frozen in a liquid nitrogen–cooled isopentane bath 7 days after treatment. Sections were fixed in 4% paraformaldehyde for 5 minutes (excluding p-JNK, HSP27, and HSP70) followed by blocking with 1% BSA (R&D Systems, DY995) or blocking buffer from the Mouse-on-Mouse Detection Kit (Vector Labs, BMK-2202) when using mouse primary antibodies for 1 hour at room temperature. GLUT1 IF images were fixed in ice-cold acetone for 5 minutes. The primary antibody was applied overnight at 4°C with the optimized dilution. The following antibodies were used: LAMA1 (Aviva Systems Biology, OACD05111; 1:200), ITGA7 (R&D Systems, MAB3518; 1:300), ITGB1 (BD Pharmingen, 557355; 1:250), αDG (IIHC-C4; 1:50, gifted by Kevin Campbell, University of Iowa, Iowa City, Iowa, USA), SGCA (DSHB, IVD3, 1:25), UTR (DSHB, MANCHO7; 1:100), p-JNK (Abcam, ab124956; 1:200), HSP27 (ProteinTech, 18284-I-AP; 1:750), HSP70 (ProteinTech, 25405-I-AP; 1:750), and GLUT1 (Cell Signaling Technology, E4S6I; 1:50). Primaries were then followed by secondary antibodies with Alexa Fluor 488 (Thermo Fisher Scientific; 1:1000), targeting the host species of the primary antibodies, incubated for 1 hour at room temperature. Wheat germ agglutinin (WGA) staining was performed to visualize the muscle fiber membrane (Thermo Fisher Scientific, WGA-AF647; 1:100) for 10 minutes. Slides were imaged using the Leica Stellaris 8 STED microscope and analyzed using ImageJ win64 software (NIH).

### Western analysis.

Gastrocnemius tissue was collected from WT controls and *dy^W–/–^* mice treated with PBS and rhLAM-111 in the contralateral leg. Tissue was immediately flash-frozen using liquid nitrogen–cooled isopentane. Proteins were extracted with T-PER Extraction Reagent (Thermo Fisher Scientific, 78510) and Halt Protease Inhibitor Cocktail (Thermo Fisher Scientific, 78430; 1:100) using the gentleMACS dissociator (Miltenyi Biotec), and left on ice for 30 minutes. Supernatants were collected, and protein concentrations were determined with Pierce BCA Protein Assay Kit (Thermo Fisher Scientific, 23225). Extracted muscle proteins (40 μg) were separated in 4%–20% Novex Tris-Glycine Mini Protein Gels (Thermo Fisher Scientific, XP04200BOX) at 200 V for 60 minutes and transferred to iBlot 2 nitrocellulose membranes (Thermo Fisher Scientific, IB23002) using iBlot 2 Western Blot Transfer System (Thermo Fisher Scientific). Membranes were blocked using OneBlock Western Blocking Buffer (Prometheus, 20-314) and incubated with primary antibodies ITGA7 (1:1000; B2-76, rabbit polyclonal produced by the Burkin lab using previously described methods; ref. 83), ITGB1 (BD Pharmingen, 557355; 1:1000) αDG (DSHB, IIH6-C4; 1:200), SGCA (Abcam, ab189254; 1:1000), HSP70 (ProteinTech, 25405-I-AP; 1:1000), and GLUT1 (Cell Signaling Technology, E4S6I; 1:500) at 4°C overnight. ITGA7 and ITGB1 were detected using secondary antibodies targeting the specific host antibodies with Alexa Fluor 800 (Thermo Fisher Scientific; 1:1000). SGCA, HSP70, and GLUT1 were detected using anti-rabbit IgG, HRP-linked antibody (Cell Signaling Technology, 7074; 1:1000), and chemiluminescent signal was developed using SuperSignal West Pico PLUS Chemiluminescent Substrate kit (Thermo Fisher Scientific, 34577). For αDG (IIHC-C4; 1:200) Western blots, primary, secondary, and 2% milk blocking buffer were diluted in 75 mM Tris-buffered saline. Bands were detected using anti-mouse IgM-HRP (Thermo Fisher Scientific, PA1-84383; 1:1000) and the previously listed chemiluminescent kit. Blots were imaged on iBright FL1500 system (Thermo Fisher Scientific, A44241). Band intensity was calculated using iBright analysis software and normalized to total protein staining.

### NADH-TR diaphorase histochemistry.

Visualization of NADH in *dy^W–/–^* and WT TA was performed using slide-mounted fresh-frozen mouse TA sectioned at 10 μm thickness. Slides were incubated in Coplin jars containing an 8 mg/5 mL NADH solution made up in Tris buffer (0.05 M, pH 7.6) and 10 mg nitroblue tetrazolium (NBT) solution for 15 minutes at 37°C followed by a wash with deionized H_2_O. Unbound NBT was then removed with increasing concentrations (30%, 60%, 90%) of acetone, after which the sections were rehydrated with decreasing concentrations of acetone. Sections were then washed with deionized H_2_O and mounted using Depex mounting media (Thermo Fisher Scientific, 50-980-372).

### Shadow rotary imaging.

Electron microscopy was performed by the rotary shadowing technique as described in Hendricks et al. (84). Briefly, protein (25–50 g/mL) in 0.2 M ammonium acetate, pH 7.4, was mixed with an equal volume of glycerol and sprayed using an atomizer onto freshly cleaved mica sheets. These were immediately transferred to a Denton 502B vacuum evaporator and dried in high vacuum, rotary shadowed with platinum/carbon at an angle of 9° (84).

### Coomassie staining.

Reduced and non-reduced rhLAM-111 (10 μg each) were run in Novex 4%–20% Tris-Glycine Plus WedgeWell gels (Thermo Fisher Scientific, XP04200BOX) with 2× Laemelli Sample Buffer (Bio-Rad, 1610737) at 100 V until the Spectra Multicolor High Range Protein Ladder (Thermo Fisher Scientific, 26625) fully ran down the length of the gel. Gels were then stained using a standard Coomassie blue staining protocol (85). Gels were imaged using iBright Imaging System (Thermo Fisher Scientific, FL1500).

### Pathway analysis.

Once data were acquired, quality control and data normalization were performed using the GeoMx DSP analysis suite following the guidelines provided by NanoString. Readouts of the human and mouse data were in counts normalized to the geometric mean of negative control proteins (rabbit IgG, rat IgG2a, and rat IgG2b), chosen based on correlation among each other. Normalized data were then downloaded to Microsoft Excel for subsequent downstream analysis.

To determine which proteins were differentially expressed, normalized counts were then processed in R and analyzed with the limma package (https://bioconductor.org/packages/release/bioc/html/limma.html). DEPs for all patient versus control (*n* = 6) spatial proteomics counts were defined as log_2_FC of 0.25 or greater and a *P* value of less than 0.05. DEPs for age-sorted data (*n* = 3) were defined as log_2_FC of 0.25 or greater and a *P* value of less than 0.1. Due to small sample sizes in human and mouse spatial proteomics and the exploratory nature of this study, a *P*-value threshold of less than 0.1 was used rather than the conventional *P* value of less than 0.05. An unpaired, 2-tailed *t* test was used to calculate and determine *P* values per protein between the 2 groups.

Subsequently, pathway enrichment analysis was conducted on the DEPs using GO annotations. Enrichment analysis was performed using clusterProfiler (https://bioconductor.org/packages/release/bioc/html/clusterProfiler.html), focusing on Biological Process ontologies. Enriched GO terms were identified based on proteins with a log_2_FC of 0.25 or greater. Upregulated and downregulated proteins were analyzed separately.

### Statistics.

All data are presented as mean ± SEM. Comparisons between multiple groups for Western blot analysis were performed by 1-way analysis of variance (ANOVA) for parametric data. Tukey’s multiple-comparison test was used for pairwise comparison. All calculations were made using GraphPad Prism 10. A *P* value of less than 0.05 was considered statistically significant.

### Study approval.

All experiments involving mice were performed under an approved protocol from the University of Nevada, Reno Institutional Animal Care and Use Committee under protocol 20-08-1071.

Deidentified human skeletal muscle tissues were provided by the Iowa Wellstone Muscular Dystrophy Specialized Research Center under their University of Iowa IRB-approved protocol (ID 200510769).

### Data availability.

The data supporting the findings of this study are available in Dryad (doi.org/10.5061/dryad.j0zpc86qw). Values for all data points in graphs are reported in the Supporting Data Values file. Additionally, data can be requested through the corresponding author.

## Author contributions

HJH, DJB, and RDW conceived and planned the experiments. DJB provided funding and overall supervision of the project. MD provided animal care. MD and HJH treated animals and collected mouse tissues. HJH, AM, LLP, and LTB carried out experiments. SAM provided and contributed to human sample preparation; he assisted in editing the manuscript. LLP and AM contributed to mouse sample preparation. HJH, LLP, and LTB performed IF staining and imaging. HJH and RDW analyzed data. PCG and HJH performed Western blot experiments and analysis. HJH performed pathway analysis and any R-related analysis. HJH, RDW, and DJB contributed to the interpretation of the results. HJH drafted the manuscript. DJB and RDW provided draft input and editing. All authors provided critical feedback and helped shape the research, analysis, and manuscript.

## Funding support

This work is the result of NIH/NINDS funding, in whole or in part, and is subject to the NIH Public Access Policy. Through acceptance of this federal funding, the NIH has been given a right to make the work publicly available in PubMed Central.

- NIH/NNDS grant 1R01NS136281-01 to DJB.
- Cure CMD award 1018515 to DJB.
- University of Iowa Paul D. Wellstone Muscular Dystrophy Specialized Research Center NIH grant P50NS053672 to SAM.

## Supplementary Material

Supplemental data

Unedited blot and gel images

Supporting data values

## Figures and Tables

**Table 1:**
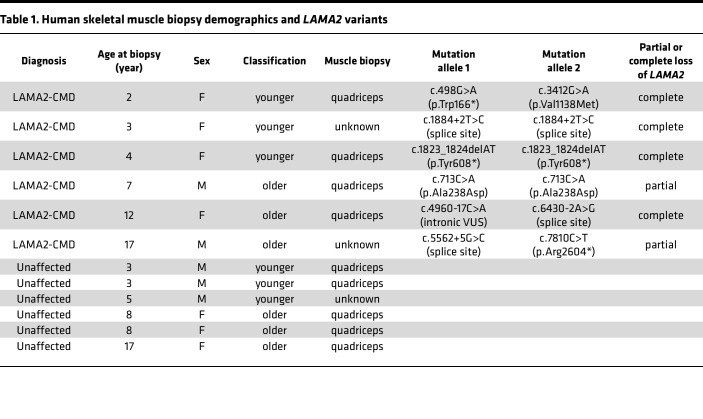
Human skeletal muscle biopsy demographics and *LAMA2* variants

**Table 2:**
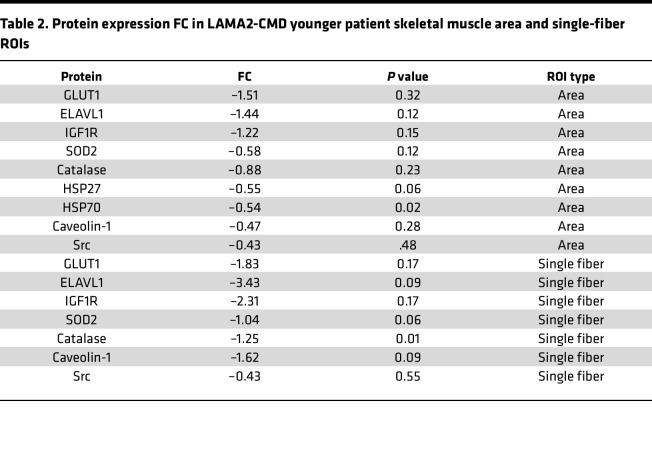
Protein expression FC in LAMA2-CMD younger patient skeletal muscle area and single-fiber ROIs

## Version 1 10/21/2025

In-Press Preview

## Version 2 11/24/2025

Electronic publication
