# Supplementary material for: Spatial proteomics reveals recombinant human laminin-111 restores adhesion signaling to laminin-α2–deficient muscle
Source: JCI Insight. 2025 Oct 16;10(22):e194581. doi: 10.1172/jci.insight.194581 (PMC12643501; doi:10.1172/jci.insight.194581)
Supplement: Supplemental data [file jciinsight-10-194581-s166.pdf]

Table S1. Excel spreadsheet containing normalized counts of 575 proteins in the GeoMX IPA panel for all human ROIs related to values used to generate heat maps and DEPs in figure 1.

Data too large to fit in PDF.

Table S2. Excel spreadsheet containing DEP values for human area and single-fiber ROIs used to generate volcano plots in figure 1.

Data too large to fit in PDF.

Table S3. Excel spreadsheet containing younger cohort age-sorted DEP values for area and single-fiber ROIs used to generate volcano plots in figure 2e-f.

Data too large to fit in PDF

Table S4. Excel spreadsheet containing older cohort age-sorted DEP values for area and single-fiber ROIs used to generate volcano plots in figure 2g-h.

Data too large to fit in PDF.

Table S5. Excel spreadsheet containing normalized counts of 60 proteins from GeoMx Mouse immuno-oncology panel for all ROIs and treatment groups used to generate DEPs displayed in figures 3b-c and 4b-c.

Data too large to fit in PDF.

Table S6. Fold Changes of Histone H3 and post-translational modification sites in younger patient area ROIs.

Histone.H3.(tri.methyl.K9)	-0.934
Histone.H3.(mono.methyl.K4)	-0.716
Histone.H3.(phospho.S28)	-0.543
Histone.H3.(di.methyl.K79)	-0.478
Histone.H3.(acetyl.K18)	-0.442
Histone.H3	-0.383
Histone.H3.(acetyl.K27)	-0.351
Histone.H3.(di.methyl.K4)	-0.334
Histone.H3.(acetyl.K9)	-0.233
Histone.H3.(acetyl.K14)	-0.201
Histone.H3.(phospho.S10)	-0.178
Histone.H3.(tri.methyl.K36)	0.115
Histone.H3.(tri.methyl.K27)	0.241

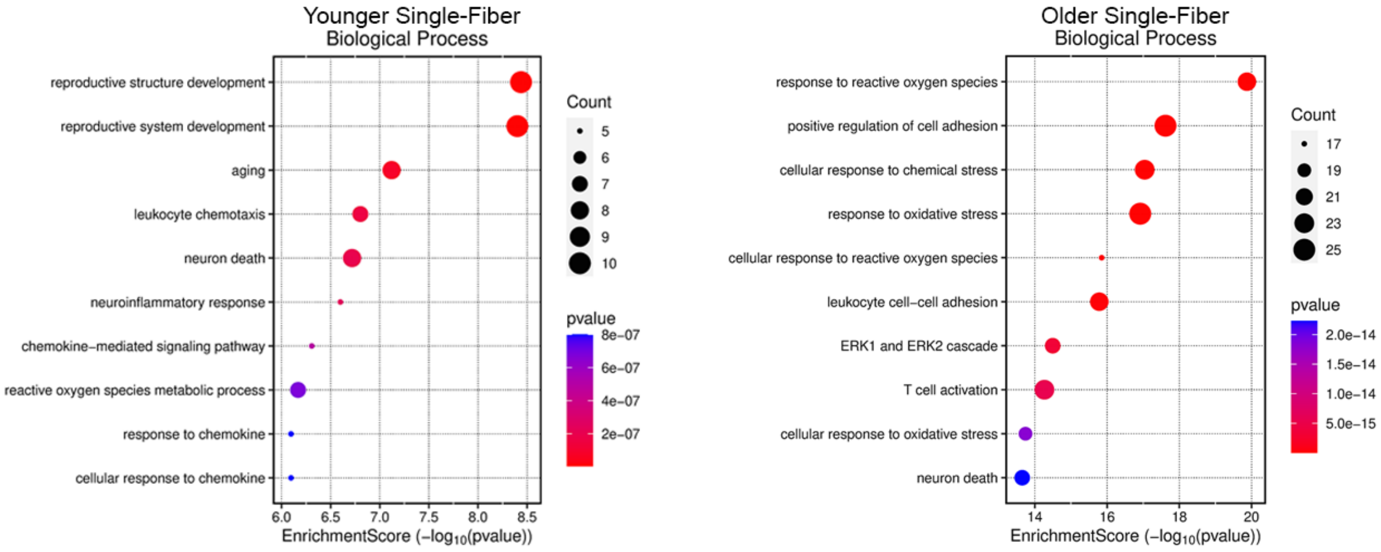

Figure S1. Additional GO Pathway analysis biological process dot plots of upregulated proteins from younger and older patient single-fiber ROIs. The top 10 biological processes derived from GO Pathway analysis from upregulated proteins in LAMA2-CMD patients including ROS and oxidative stress responses.

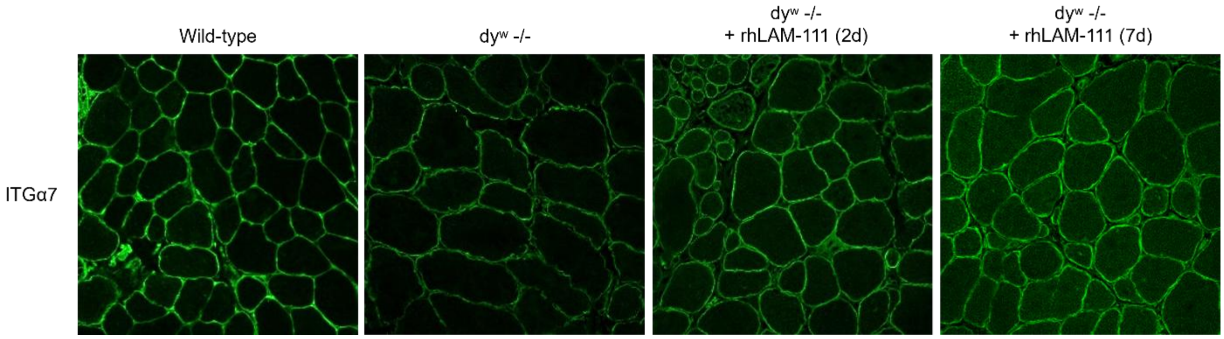

Figure S2. ITGa7 IF displaying increased localization 2 and 7 days post intramuscular injections of rhLAM-111.

Transverse sections of whole tibialis anterior muscle from 5-week-old WT, dy^{W}-/- and dy^{W}-/- post 2 and 7 days rhLAM-111 treatment with antibody against ITGa7. Antibodies were visualized using indirect fluorescence microscopy. Imaged on Leica-Sp8 at 40X magnification, scale bar = 50µm.

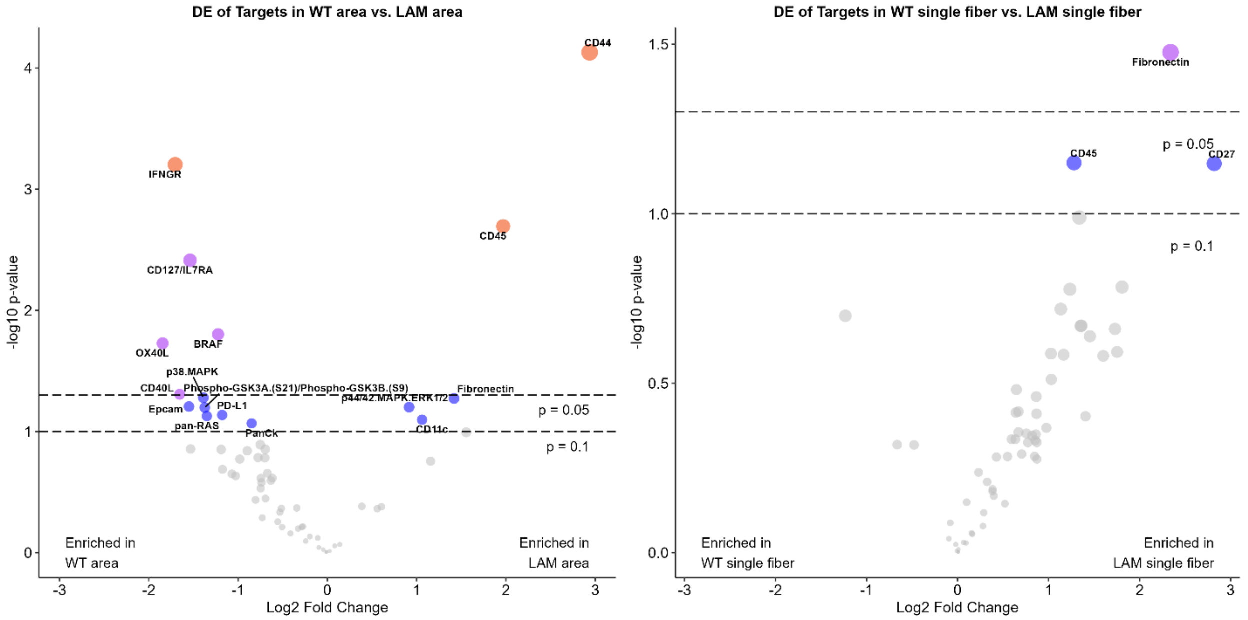

Figure S3. Volcano plots displaying DEPs between WT and LAM in area and single-fiber ROIs. Area and single-fiber ROI DEPs volcano plot for WT compared to rhLAM-111 treated dy^{W}-/-, respectively. Upper dashed line indicates p-value = 0.05 and lower dashed line indicates p-value = 0.1.
