# Supplementary material for: Spatial proteomics reveals recombinant human laminin-111 restores adhesion signaling to laminin-α2–deficient muscle
Source: JCI Insight. 2025 Oct 16;10(22):e194581. doi: 10.1172/jci.insight.194581 (PMC12643501; doi:10.1172/jci.insight.194581)

Figure 6B. Recombinant human laminin-111 separated on 4-20% SDS-Page gel, cropped region shown in manuscript

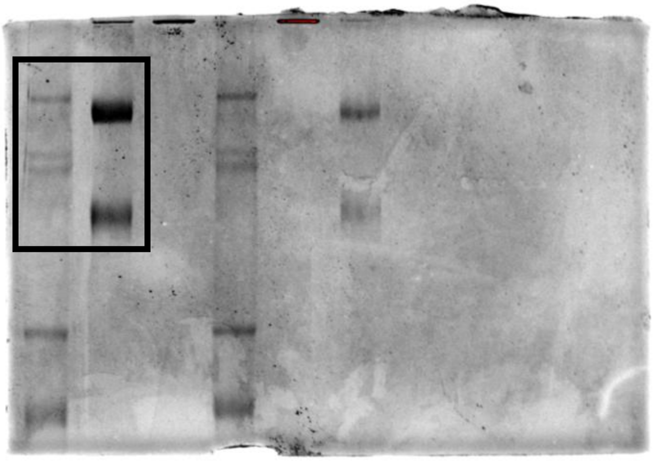

Figure 7B. Integrin-α7 fluorescent WB, primary B2-76 (Burkin lab, 1:1000)

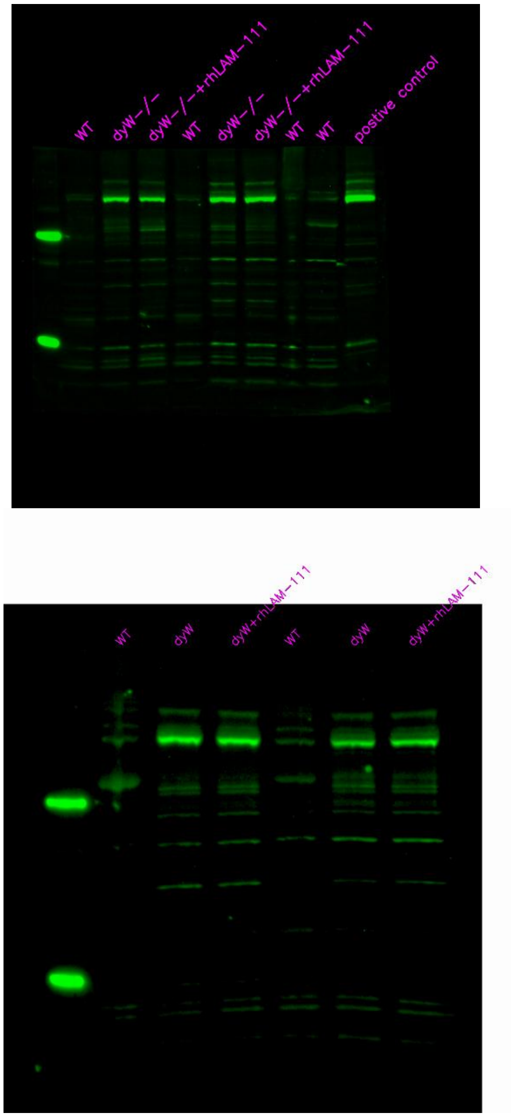

Figure 7B. Integrin-β1 fluorescent WB, primary 557355 (BDPharmingen, 1:1000)

*These lanes were testing different protein concentrations and was not used in this study

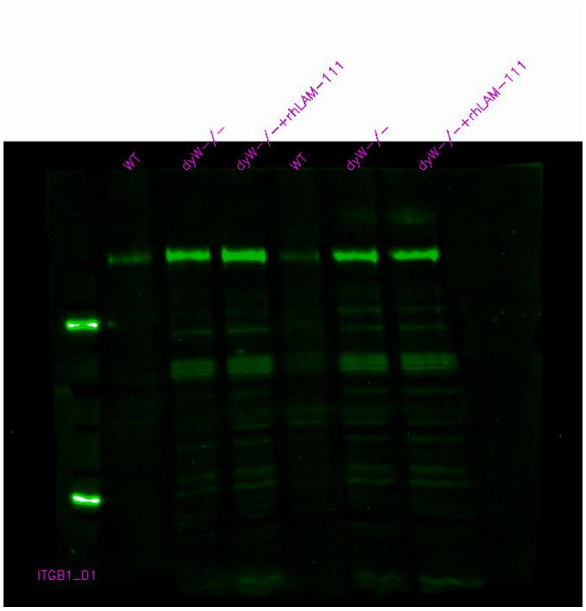

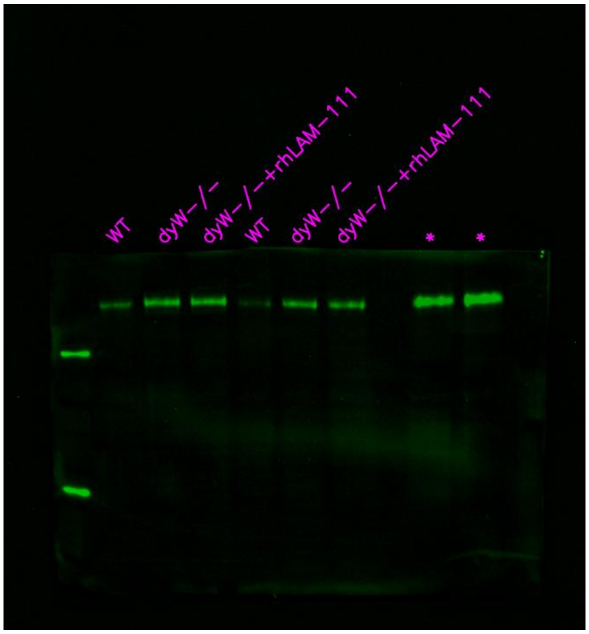

Figure 7B. Alpha-dystroglycan chemiluminescent WB, primary: IIH6-C4 (DSHB, 1:200)

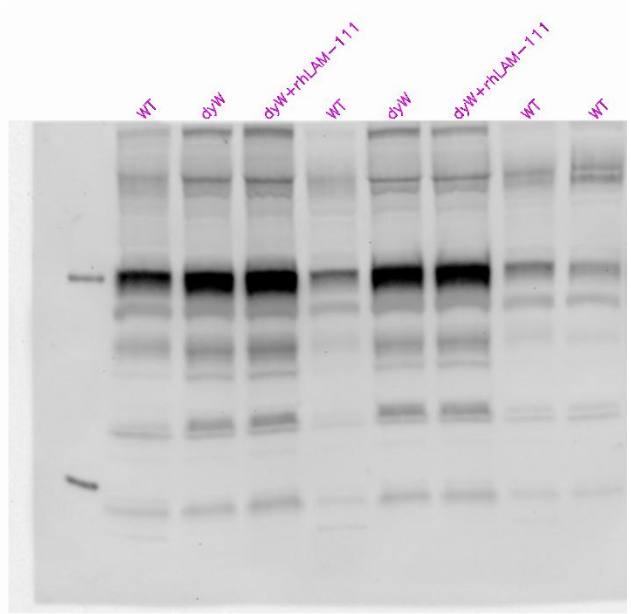

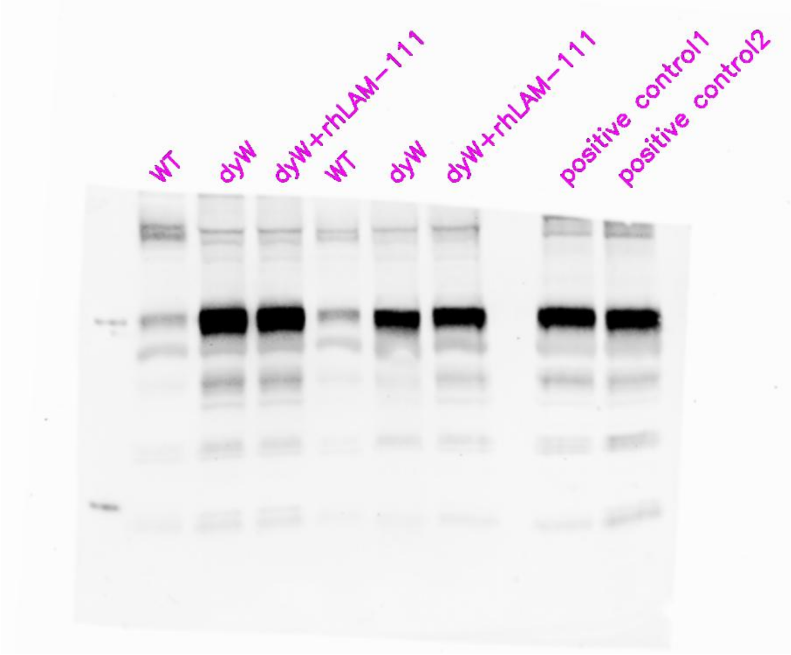

Figure 7B. Alpha-sarcoglycan chemiluminescent WB, primary: AB189254 (Abcam, 1:1000)

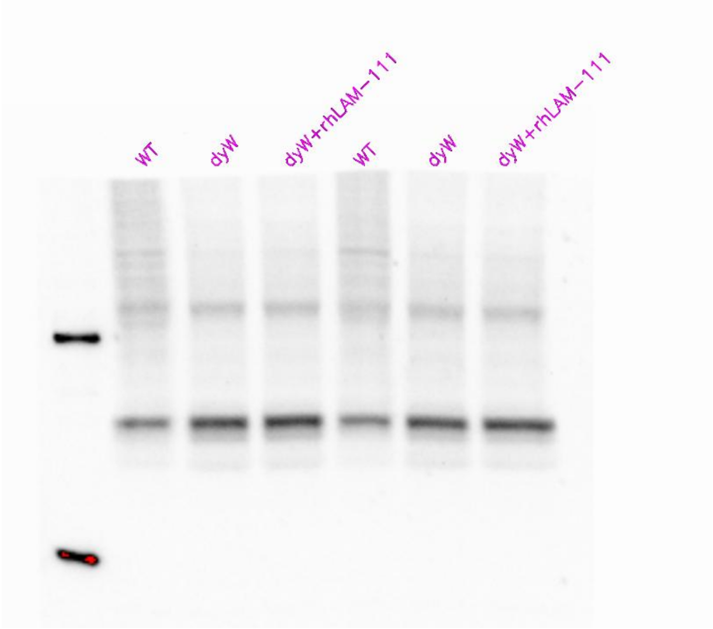

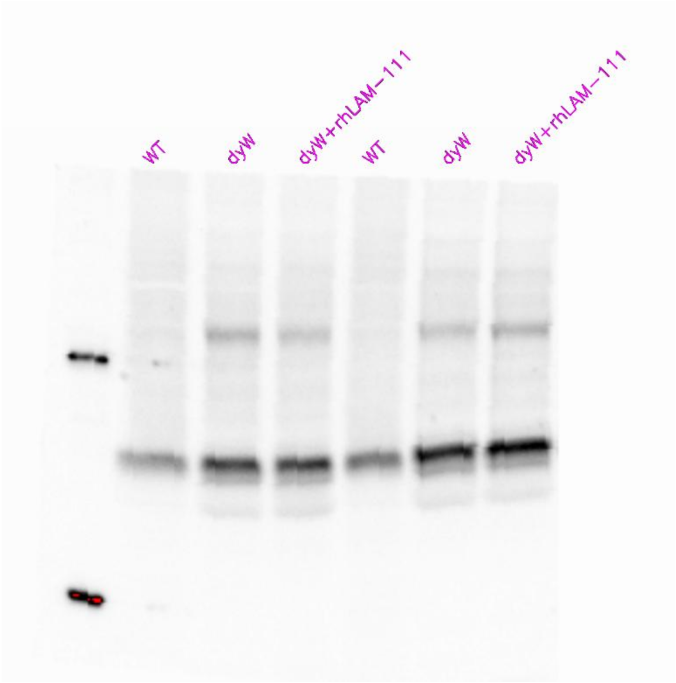

Figure 8B. HSP70 chemiluminescent WB, 25405-I-AP (ProteinTech, 1:1000), cropped region shown in manuscript

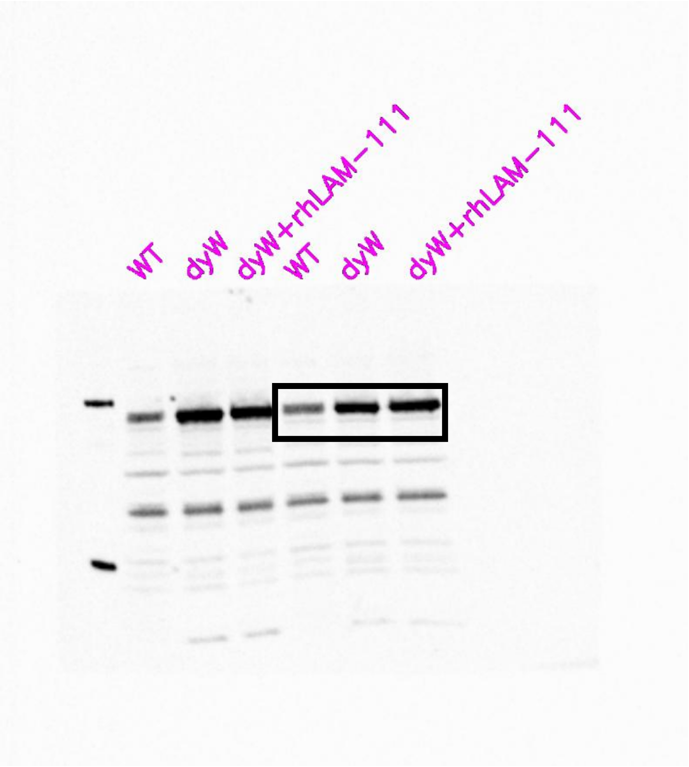

Figure 8C. GLUT1 chemiluminescent WB, E4S6I (Cell Signaling, 1:500), cropped region shown in manuscript

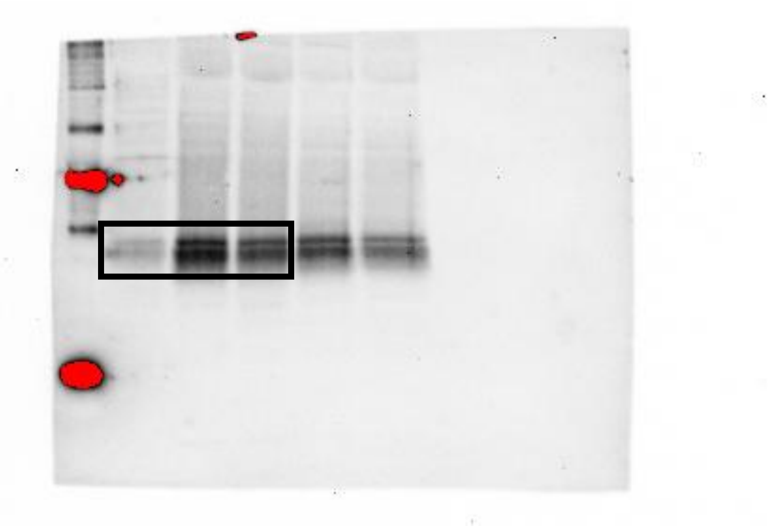

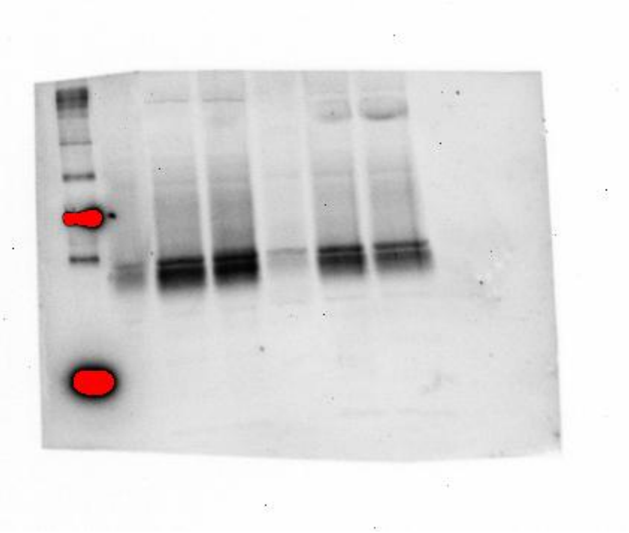

Supplement: Unedited blot and gel images [file jciinsight-10-194581-s167.pdf]
